# Tissue regeneration strategies based on mesenchymal stem cell-derived extracellular vesicles: from bench to bedside

**DOI:** 10.1093/burnst/tkag030

**Published:** 2026-05-23

**Authors:** Shi-Han Mu, Rang Li, Peng-Fei Wang, Ying-Shuang Shi, Yu-Wei Zhou, Yan Jin, Fang Jin, Bing-Dong Sui

**Affiliations:** State Key Laboratory of Oral & Maxillofacial Reconstruction and Regeneration, National Clinical Research Center for Oral Diseases, Shaanxi International Joint Research Center for Oral Diseases, Center for Tissue Engineering, School of Stomatology, Fourth Military Medical University, 145 West Changle Road, Xincheng District, Xi’an, Shaanxi 710032, China; State Key Laboratory of Oral & Maxillofacial Reconstruction and Regeneration, National Clinical Research Center for Oral Diseases, Shaanxi Clinical Research Center for Oral Diseases, Department of Orthodontics, School of Stomatology, Fourth Military Medical University, 145 West Changle Road, Xincheng District, Xi’an, Shaanxi 710032, China; State Key Laboratory of Oral & Maxillofacial Reconstruction and Regeneration, National Clinical Research Center for Oral Diseases, Shaanxi International Joint Research Center for Oral Diseases, Center for Tissue Engineering, School of Stomatology, Fourth Military Medical University, 145 West Changle Road, Xincheng District, Xi’an, Shaanxi 710032, China; State Key Laboratory of Oral & Maxillofacial Reconstruction and Regeneration, National Clinical Research Center for Oral Diseases, Shaanxi Key Laboratory of Stomatology, Department of Operative Dentistry and Endodontics, School of Stomatology, Fourth Military Medical University, 145 West Changle Road, Xincheng District, Xi’an, Shaanxi 710032, China; State Key Laboratory of Oral & Maxillofacial Reconstruction and Regeneration, National Clinical Research Center for Oral Diseases, Shaanxi International Joint Research Center for Oral Diseases, Center for Tissue Engineering, School of Stomatology, Fourth Military Medical University, 145 West Changle Road, Xincheng District, Xi’an, Shaanxi 710032, China; State Key Laboratory of Oral & Maxillofacial Reconstruction and Regeneration, National Clinical Research Center for Oral Diseases, Shaanxi Clinical Research Center for Oral Diseases, Department of Orthodontics, School of Stomatology, Fourth Military Medical University, 145 West Changle Road, Xincheng District, Xi’an, Shaanxi 710032, China; School of Basic Medicine, Fourth Military Medical University, 169 West Changle Road, Xincheng District, Xi’an, Shaanxi 710032, China; State Key Laboratory of Oral & Maxillofacial Reconstruction and Regeneration, National Clinical Research Center for Oral Diseases, Shaanxi International Joint Research Center for Oral Diseases, Center for Tissue Engineering, School of Stomatology, Fourth Military Medical University, 145 West Changle Road, Xincheng District, Xi’an, Shaanxi 710032, China; State Key Laboratory of Oral & Maxillofacial Reconstruction and Regeneration, National Clinical Research Center for Oral Diseases, Shaanxi Clinical Research Center for Oral Diseases, Department of Orthodontics, School of Stomatology, Fourth Military Medical University, 145 West Changle Road, Xincheng District, Xi’an, Shaanxi 710032, China; School of Basic Medicine, Fourth Military Medical University, 169 West Changle Road, Xincheng District, Xi’an, Shaanxi 710032, China; State Key Laboratory of Oral & Maxillofacial Reconstruction and Regeneration, National Clinical Research Center for Oral Diseases, Shaanxi International Joint Research Center for Oral Diseases, Center for Tissue Engineering, School of Stomatology, Fourth Military Medical University, 145 West Changle Road, Xincheng District, Xi’an, Shaanxi 710032, China; State Key Laboratory of Oral & Maxillofacial Reconstruction and Regeneration, National Clinical Research Center for Oral Diseases, Shaanxi Clinical Research Center for Oral Diseases, Department of Orthodontics, School of Stomatology, Fourth Military Medical University, 145 West Changle Road, Xincheng District, Xi’an, Shaanxi 710032, China; School of Basic Medicine, Fourth Military Medical University, 169 West Changle Road, Xincheng District, Xi’an, Shaanxi 710032, China; State Key Laboratory of Oral & Maxillofacial Reconstruction and Regeneration, National Clinical Research Center for Oral Diseases, Shaanxi International Joint Research Center for Oral Diseases, Center for Tissue Engineering, School of Stomatology, Fourth Military Medical University, 145 West Changle Road, Xincheng District, Xi’an, Shaanxi 710032, China; State Key Laboratory of Oral & Maxillofacial Reconstruction and Regeneration, National Clinical Research Center for Oral Diseases, Shaanxi Clinical Research Center for Oral Diseases, Department of Orthodontics, School of Stomatology, Fourth Military Medical University, 145 West Changle Road, Xincheng District, Xi’an, Shaanxi 710032, China; State Key Laboratory of Oral & Maxillofacial Reconstruction and Regeneration, National Clinical Research Center for Oral Diseases, Shaanxi International Joint Research Center for Oral Diseases, Center for Tissue Engineering, School of Stomatology, Fourth Military Medical University, 145 West Changle Road, Xincheng District, Xi’an, Shaanxi 710032, China

**Keywords:** Mesenchymal stem cells, Extracellular vesicles, Cell-free therapy, Tissue regeneration, Clinical translation, Exosomes, Microvesicles, Apoptotic vesicles

## Abstract

Regenerative medicine is undergoing a paradigm shift from live-cell therapies to cell-free strategies. Within this evolving field, mesenchymal stem cell-derived extracellular vesicles (MSC-EVs) have emerged as a leading platform. These nanoscale vesicles deliver bioactive cargo that mediates critical therapeutic functions, including immunomodulation, angiogenesis, and anti-fibrosis. Furthermore, they offer improved safety, greater potential for standardization, and enhanced scalability compared to traditional live-cell therapies. However, clinical translation remains constrained by several challenges, such as inherent vesicle heterogeneity, limited targeting specificity, and bottlenecks in large-scale manufacturing. This review systematically examines the biogenesis of MSC-EVs, focusing specifically on exosomes, microvesicles, and apoptotic vesicles. We evaluate their functional performance across diverse regeneration contexts, encompassing orofacial, barrier, musculoskeletal, and visceral tissue regeneration. We further highlight innovative engineering strategies designed to enhance therapeutic efficacy, such as surface modification, cargo loading, and biomaterial-integrated delivery systems. In addition, we introduce an emerging approach utilizing engineered MSC aggregate-derived EVs inspired by organ morphogenesis. Finally, this article details the strategic framework required for clinical translation. The framework encompasses scalable production, rigorous quality control, comprehensive non-clinical studies, evolving regulatory pathways, and the current clinical trial landscape. Collectively, this work provides an integrated roadmap for advancing MSC-EVs as a next-generation precision platform for cell-free therapeutics.

## Graphical Abstract

**Graphical Abstract:**
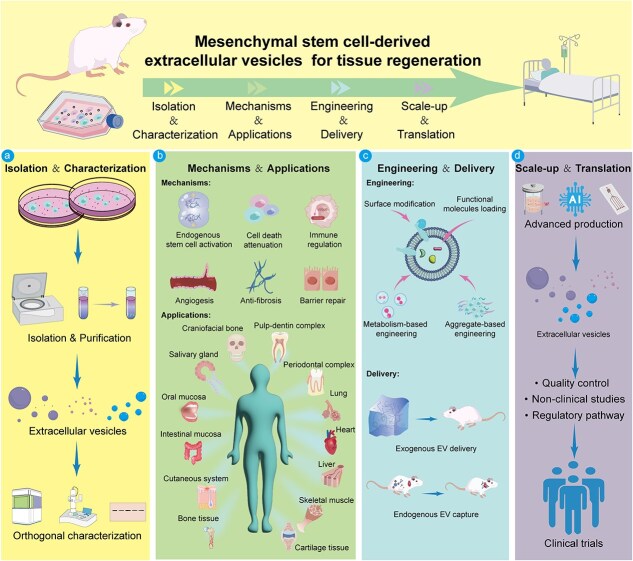

## Highlights

- The biogenesis pathways and functional heterogeneity of MSC-EVs are outlined.
- Regenerative effect and mechanisms of MSC-EVs across multiple systems are examined.
- Engineering strategies to enhance targeting and efficacy of MSC-EVs are evaluated.
- An integrated clinical translation roadmap of MSC-EVs is proposed.

## Background

Regenerative medicine is undergoing a paradigm shift from live-cell therapies toward cell-free approaches. This shift is driven by evidence that the therapeutic benefits of mesenchymal stem cells (MSCs) are mediated chiefly by paracrine mechanisms, particularly through extracellular vesicles (EVs), rather than by durable engraftment or direct tissue replacement [1–4]. Early mechanistic studies not only demonstrated the limited long-term engraftment of MSCs but also revealed that the apoptosis of infused MSCs can modulate immune responses, thereby reinforcing the paracrine hypothesis [3, 5]. This understanding has prompted discussions regarding a proposed name change to better reflect their biological role as medicinal signaling cells rather than traditional stem cells. Despite this shift in perspective, MSC-based live-cell therapeutics continue to face significant challenges. Notably, donor- and process-related batch variability complicates the precise definition of cellular identity and potency. This variability persists even following the establishment of critical quality attributes (CQAs) by the International Society for Cell and Gene Therapy [6, 7]. Additional limitations encompass infusion-related risks, such as tissue factor-mediated instant blood-mediated inflammatory reaction and complement/coagulation activation. Furthermore, there is a high degree of complexity and cost associated with Good Manufacturing Practice (GMP)-compliant manufacturing and lot-release testing [6, 7]. As a result, MSC-derived EVs (MSC-EVs) are increasingly regarded as the key paracrine effectors, marking a promising transition to cell-free regenerative strategies [8].

EVs are nanoscale, lipid-bilayer enclosed particles secreted by nearly all cell types. They transport proteins, lipids, and nucleic acids to facilitate intercellular communication across diverse tissues [9]. Their inherent biocompatibility, ability to traverse biological barriers, and non-replicative nature make them highly attractive as therapeutic platforms [10, 11]. Furthermore, the field is becoming increasingly standardized by community guidelines, specifically the Minimal Information for Studies of Extracellular Vesicles (MISEV2023). These guidelines establish rigorous criteria for EV isolation, characterization, and reporting, thereby enhancing reproducibility and supporting their translation into well-defined biological products [12, 13]. The MISEV2023 guideline also informs the nomenclature framework used in this review, wherein generic terms (EVs), size-based descriptors (small EVs, sEVs; large EVs, lEVs), and biogenesis-specific terms (exosomes, EXOs; microvesicles, MVs; apoptotic vesicles, ApoVs) are applied according to defined evidence thresholds, as detailed in Section 2.1.1. Collectively, these properties endow EVs with superior safety, controllability, and scalability compared to traditional live-cell therapies, establishing them as a next-generation regenerative treatment platform.

Among various EV sources, MSC-EVs hold a particularly distinguished position for regenerative applications. This prominence originates from the unique biology of their parent cells. MSCs inherently express low levels of major histocompatibility complex class II (MHC II) molecules and secrete a broad spectrum of immunomodulatory factors. Consequently, MSC-EVs inherit a profile of low immunogenicity coupled with multidimensional regenerative capacities, including immunomodulation, pro-angiogenesis, and cytoprotection [8, 14, 15]. In addition to these benefits, MSC-EVs possess distinct advantages over EVs derived from other stem cell sources. For instance, induced pluripotent stem cell-derived EVs (iPSC-EVs) carry potential risks of genomic instability associated with somatic reprogramming [16, 17]. Meanwhile, the clinical development of embryonic stem cell-derived EVs (ESC-EVs) remains constrained by persistent ethical controversies [18]. This distinct profile of safety and ethical acceptance positions MSC-EVs more advantageously than other stem cell-derived EVs.

The advantages of MSC-EVs, however, extend beyond these general characteristics to include remarkable functional heterogeneity rooted in their tissue of origin. A growing body of multi-omics evidence reveals that the tissue source of MSCs imprints distinct molecular signatures on their EVs, which influence, rather than strictly determine their bioactivity [19]. For instance, bone marrow-derived MSC-EVs (BMMSC-EVs) often carry osteo-angiogenic regulators reflective of the marrow’s skeletal and hematopoietic niche, enhancing bone repair in preclinical models [20]. Umbilical cord-derived MSC-EVs (UCMSC-EVs) are enriched with immunomodulatory factors consistent with their role in fetomaternal immune tolerance, rendering them particularly suitable for immunoregulatory applications [21, 22]. Adipose-derived MSC-EVs (ADMSC-EVs), in turn, exhibit matrix-remodeling signatures aligned with their soft-tissue origin and effectively promote cutaneous wound healing [23]. Collectively, these distinctions underscore why MSC-EVs are increasingly regarded as the EV platform of choice for regenerative medicine, with special emphasis on tissue-specific subsets.

This review comprehensively synthesizes recent advances in MSC-EV research and tracks their translational progression from fundamental biology to clinical applications. We begin by systematically delineating MSC-EV biogenesis, characterization, and functional heterogeneity. Clear distinctions are made among EXOs, MVs, and ApoVs to establish a robust foundation for subsequent analyses. We then critically evaluate the regenerative efficacy of MSC-EVs across diverse tissue systems, including orofacial, barrier, musculoskeletal, and visceral tissues. During this evaluation, we focus specifically on shared mechanistic principles identified throughout the literature. A dedicated section analyzes innovative engineering strategies, such as surface modification, cargo loading, and biomaterial-assisted delivery systems, all of which are designed to overcome inherent heterogeneity and enhance targeting precision. Furthermore, we examine an emerging paradigm inspired by organ morphogenesis, which relies on engineered MSC aggregate-derived EVs. Finally, the review outlines the clinical translation pathway for MSC-EVs. We address the key challenges of scalable production, artificial intelligence (AI)-optimized manufacturing, rigorous quality control (QC), non-clinical studies, evolving regulatory frameworks, and the current clinical trial landscape. Collectively, this work provides an integrated roadmap for advancing MSC-EVs as precise, safe, and effective cell-free therapeutics in regenerative medicine.

## Review

### Biogenesis and key attributes of MSC-EVs

#### General MSC-EVs considerations

The terminology for EVs in the MSC field exhibits heterogeneity and is frequently influenced by isolation and measurement methodologies. To ensure clarity, rigor, and cross-study comparability, the consistent adoption of generic EV nomenclature supplemented by explicit operational qualifiers is essential [12, 24]. Therefore, we employ ‘EVs’ as the umbrella term with descriptors based on measurable properties, such as size (*e.g.* sEVs and lEVs, defined within method-specific size ranges), cellular origin, density, or biochemical composition. Biogenesis-specific labels (*e.g.* EXOs, MVs, or ApoVs) are avoided unless subcellular provenance is rigorously demonstrated through multimodal evidence (*e.g.* live-cell imaging, selective genetic/pharmacologic perturbation, topology and density/size fractionation) [12].

**Figure 1:**
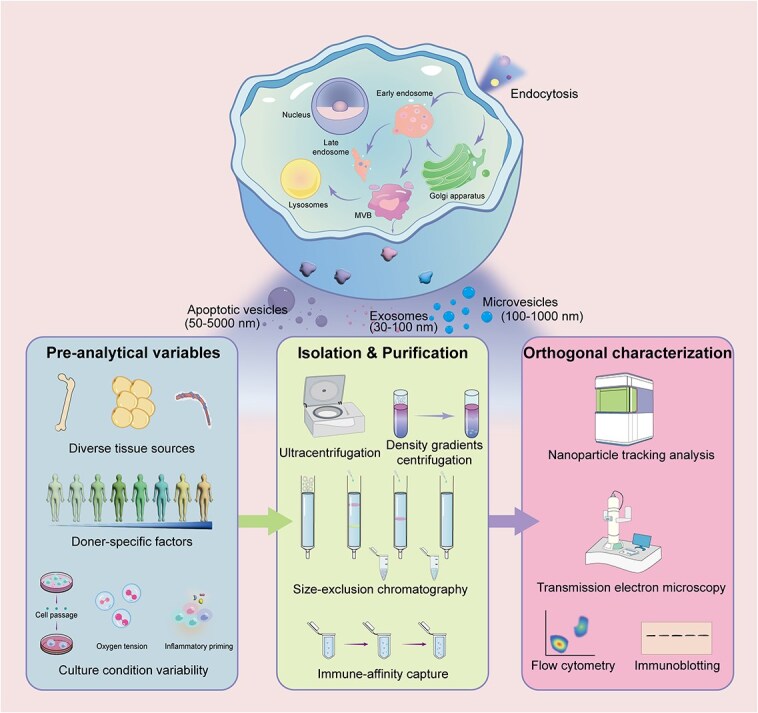
Major subtypes and production pipeline of MSC-EVs. This schematic summarizes three major MSC-EV subtypes and their standardized production pipeline. The subtypes are exosomes (endosome-derived, 30–100 nm), microvesicles (plasma membrane-budded, 100–1000 nm), and apoptotic vesicles (generated during programmed cell death, 50–5000 nm). The pipeline spans from pre-analytical variable control through isolation and purification to orthogonal characterization. *MVB* multivesicular body, *TNF-α* tumor necrosis factor-alpha, *IFN-γ* interferon-gamma

Pre-analytical variables exert substantial and frequently underappreciated effects on MSC-EV yield, composition, and function (Figure 1). For MSCs specifically, these factors serve as critical modulators that can be leveraged to educate cells and therapeutically tailor the resulting EV output. Essential variables include donor variability and tissue source. For instance, bone marrow, adipose tissue, or umbilical cord derivations confer distinct molecular signatures and functional biases to the EVs [20–23]. Furthermore, culture conditions act not as trivial protocol details but as active determinants of EV potency. These critical conditions include passage number, oxygen tension (wherein hypoxia typically enhances pro-angiogenic cargo), and inflammatory priming with cytokines such as interferon-gamma (IFN-γ) or tumor necrosis factor-alpha (TNF-α) to boost immunomodulatory properties [25, 26]. Additionally, culture medium formulation (including serum supplementation), cell confluency, and conditioned medium storage or handling require rigorous standardization. These parameters should be systematically documented to enable reproducible cross-study and longitudinal comparisons [12,27–29]. Crucially, serum-derived contaminants such as lipoproteins and protein aggregates may alter EV biogenesis and co-isolate with EVs. This necessitates the use of serum pre-clearing or EV-depleted supplements, paired with the transparent reporting of depletion protocols [12,27–30].

No single isolation method currently yields an absolutely pure EV population. Instead, each approach presents characteristic trade-offs between recovery and specificity, as well as distinct co-isolation biases that should be clearly documented. Widely used techniques include ultracentrifugation, ultrafiltration, density gradient centrifugation (DGC) (*e.g.* using iodixanol or sucrose), size-exclusion chromatography (SEC), immune-affinity capture, and polymer-based precipitation. These methods vary in their tendency to co-enrich non-vesicular components, including lipoproteins, soluble protein complexes, nucleoprotein particles, and other nonvesicular extracellular nanoparticles (NVEPs) (Figure 1) [31–33]. Recent studies indicate that while ultracentrifugation is widely regarded as the gold standard for EV isolation, it suffers from moderate yields, lengthy processing times, and limited purity. The co-isolation of protein aggregates and lipoproteins is a common limitation for this technique [31–33]. In contrast, ultrafiltration is easy to perform and requires no special reagents or equipment, but it carries the risk of membrane clogging and the subsequent loss of small-diameter EVs [31–33]. SEC offers a recovery rate of 40%–80% and preserves the biological structure and function of EVs. However, lipoprotein contamination may still occur [31–33]. Polymer-based precipitation is simple and suitable for large sample volumes, but it yields low purity and may introduce polymer residues that interfere with downstream analyses [31–33]. Immune-affinity capture provides high specificity for EV subtypes. Despite this, its overall yield is highly dependent on antibody specificity, and the process may involve harsh elution conditions [31–33]. Finally, DGC achieves high purity but remains labor-intensive, time-consuming, and restricted to small sample volumes [31–33]. Therefore, the choice of isolation method should be rigorously justified in the context of the sample type and research objectives, with detailed reporting of processing parameters, yields, and critical loss steps (Table 1).

**Table 1:** Comparison of MSC-EV isolation methods

Method	Yield *vs* Specificity	Major Co-isolated contaminants	Recommended applications	Mitigation strategies
Ultracentrifugation	Moderate to High yield; moderate specificity	NVEPs with similar sedimentation coefficients (*e.g.* protein aggregates, lipoproteins)	Initial enrichment, large-volume samples	Combine with DGC or SEC to improve specificity
DGC	Low yield; high specificity	Minimal if fractions are properly collected	EV subtype analysis, high-purity requirements, removal of lipoproteins/protein aggregates	Collect specific density fractions
SEC	High yield; moderate specificity	Similarly sized particles such as lipoproteins, viruses, and protein aggregates	Functional studies, potency assays, gentle isolation	Combine with ultrafiltration for concentration; combine with DGC for higher purity
Ultrafiltration	High yield; low specificity	All particles above the MWCO, including lipoproteins, protein aggregates	Volume reduction, pre-concentration	Select appropriate MWCO; combine with SEC for purification
Immune-affinity capture	Low yield (subtype-specific); high specificity for target	Non-specifically bound materials, affinity reagents (antibodies, beads), elution components	Subtype-specific studies, biomarker validation, single-EV analysis	Use gentle elution (avoid low pH/proteases if possible)
Polymer-Based Precipitation	High yield; very low specificity	Polymers (*e.g.* PEG), lipoproteins, protein aggregates, nucleoprotein complexes	Not recommended for purity-dependent applications	Discouraged for use alone; if used, must include polymer removal step and combine with SEC/DGC

*MSC* mesenchymal stem cell, *EV* extracellular vesicle, *NVEPs* nonvesicular extracellular nanoparticles, *DGC* density gradient centrifugation, *SEC* size-exclusion chromatography, *MWCO* molecular weight cut-off, *pH* potential of hydrogen, *PEG* polyethylene glycol

Orthogonal characterization remains essential for rigorous EV analysis (Figure 1). Particle concentration and size distribution should be determined using calibrated methods such as nanoparticle tracking analysis or resistive pulse sensing, accompanied by explicit acknowledgment of instrument detection limits and underlying model assumptions. Morphology and membrane topology should be verified using transmission electron microscopy or cryo-electron microscopy. Furthermore, single-particle phenotyping and flow cytometry must adhere to established reporting guidelines such as MIFlowCyt-EV, which mandate full disclosure of instrument settings, trigger thresholds, and analysis algorithms [12, 34]. Proteomic profiling *via* liquid chromatography–tandem mass spectrometry (LC–MS/MS) and quantitative immunoblotting should confirm the enrichment of canonical EV markers alongside depletion of negative markers, including organellar proteins such as calnexin and Golgi matrix protein 130 (GM130), as well as apolipoproteins associated with lipoproteins. Small ribonucleic acid (RNA) sequencing must be conducted under conditions that effectively distinguish vesicular RNAs from non-vesicular ribonucleoprotein complexes [12]. Finally, the use of community reporting platforms such as EV-TRACK and compliance with minimal information guidelines are strongly recommended to enhance reproducibility and support future meta-analyses [12, 35].

This section has outlined the common methodological frameworks, QC criteria, and overlapping functional attributes relevant to MSC-EV research. Together, these elements form a unified structure that adheres to community-endorsed recommendations aimed at improving scientific rigor, minimizing interpretive ambiguity, and facilitating the translation of MSC-EV biology toward therapeutic applications. The following subsections will now turn specifically to EXOs, MVs, and ApoVs, elucidating their distinct biogenetic pathways and the functional properties unique to each subtype.

#### EXOs

In accordance with MISEV2023 guidelines, we employ the term ‘EXOs’ specifically to denote endosome-derived EVs (Table 2). This designation requires mechanistic evidence of intraluminal vesicle (ILV) formation within multivesicular bodies (MVBs) and subsequent MVB fusion with the plasma membrane [12, 36]. Morphologically, EXOs appear as saucer-shaped vesicles enclosed by a lipid bilayer, with a diameter ranging from 30 to 100 nm, and display a buoyant density between 1.13 and 1.19 g·ml^−1^ in sucrose gradients (Figure 1) [36]. These physical properties are conserved across EXOs from diverse cellular origins. MSC-EXOs typically conform to this size range and are enriched in canonical tetraspanins (CD63, CD81, CD9). Beyond these common exosomal markers, MSC-EXOs also express parental cell-specific surface markers, including CD29, CD44, CD73, and CD90, which serve as critical identifiers of their mesenchymal origin [37].

**Table 2:** Comparative summary of MSC-EV subtypes

Parameter	Exosomes	Microvesicles	Apoptotic vesicles
Size range	30–100 nm	100–1000 nm	50–5000 nm
Buoyant density	1.13–1.19 g·ml^−1^	1.13–1.19 g·ml^−1^	1.16–1.28 g·ml^−1^
Biogenesis pathway	Endosomal pathway: ILV formation within MVBs, followed by MVB-plasma membrane fusion	Direct outward budding and fission of the plasma membrane	Executed during apoptosis: caspase-driven membrane blebbing, apoptopodia formation, and cell fragmentation
Key triggers	Cellular activation or stress (*e.g.* 3D culture, hypoxia, microgravity, inflammatory priming, *etc.*)	Cellular activation or stress (*e.g.* Ca^2+^ influx, ATP stimulation, hypoxia, *etc.*)	Apoptosis induction (*e.g.* staurosporine, ultraviolet irradiation, nutrient deprivation, *etc.*)
Hallmark markers	Tetraspanins (CD63, CD81, CD9); MSC markers (CD29, CD44, CD73, CD90)	Phosphatidylserine exposure (often Annexin V^+^); MSC surface markers	Near-universal phosphatidylserine exposure (strong Annexin V^+^); MSC markers; apoptosis-associated proteins (*e.g.* FAS, calreticulin, *etc.*)
Cargo characteristics	Proteins, lipids, nucleic acids, and metabolites	Cytosolic components, membrane-associated molecules, and functional organelles	Chromosomal DNA, micronuclei, partially degraded organelles, lipids, proteins, and various RNA species
Evidence for subtype	Endosomal origin *via* imaging of MVB fusion, perturbation of EXO biogenesis pathways (ESCRT-dependent/independent), or endosomal marker co-fractionation	Direct plasma membrane budding *via* imaging, Ca^2+^-dependent lipid scrambling, calpain activation, or ARF6/ROCK pathway perturbation	Caspase-dependent generation *via* apoptosis induction, caspase activity assays, and imaging of membrane blebbing or apoptopodia

*MSC* mesenchymal stem cell, *EV* extracellular vesicle, *ILV* intraluminal vesicle, *MVBs* multivesicular bodies, *ATP* adenosine triphosphate, *FAS* apoptosis-mediating surface antigen FAS, *RNA* ribonucleic acid, *ESCRT* endosomal sorting complex required for transport, *ARF6* ADP-ribosylation factor 6, *ROCK* Rho-associated protein kinase

Within this framework, prospective exosomal cargoes must first be delivered to early endosomes prior to MVB maturation. These cargoes arrive either *via* endocytosis from the plasma membrane or through anterograde traffic from the trans-Golgi network before entering the ILV-forming domain of the MVB [9] (Figure 1). EXO biogenesis then proceeds *via* two broad routes: an endosomal sorting complex required for transport (ESCRT)-dependent pathway and, in parallel, ESCRT-independent mechanisms. Both pathways are conserved in mammalian systems and are relevant to MSCs, as their EXOs carry typical endosomal markers and reflect conserved cargo-sorting patterns [9, 38].

In ESCRT-dependent biogenesis, ESCRT-0 and ESCRT-I initially cluster ubiquitinated transmembrane cargoes within microdomains of the MVB limiting membrane [9]. ESCRT-II subsequently recruits ESCRT-III, which mediates membrane budding and scission to generate ILVs [9]. Additionally, a specialized syndecan/syntenin/apoptosis-linked gene 2-interacting protein X (ALIX) axis serves to link specific cargoes to the ESCRT-III subunit charged multivesicular body protein 4. This specific pairing facilitates ILV formation directed toward secretion rather than lysosomal degradation. Consequently, this axis represents a central mechanism for EXO biogenesis from endosomes in MSCs [39]. In contrast, ESCRT-independent mechanisms rely on lipid- and tetraspanin-organized microdomains. For example, ceramide generated by neutral sphingomyelinase-2 can induce negative membrane curvature to promote inward budding. Simultaneously, tetraspanins such as CD63, CD81, CD82, and CD9 form platforms that coordinate cargo selection and facilitate membrane remodeling [9, 40].

Following their formation, MVBs containing ILVs undergo a critical fate determination. These organelles face two mutually exclusive destinies: fusion with lysosomes, resulting in the degradation of ILVs, or fusion with the plasma membrane, leading to the extracellular release of ILVs as EXOs [9] (Figure 1). When the latter route is activated, the release mechanism functions distinctly from ILV biogenesis. This secretory process proceeds through three sequential stages: (i) outward transport of MVBs along cytoskeletal tracks toward the cell periphery, a process in which the small GTPase Rab27b participates by helping to keep MVBs in a peripheral position; (ii) docking of peripheral MVBs to the plasma membrane, mediated in part by Rab27a through remodelling of the actin cortex; and (iii) fusion of the MVB membrane with the plasma membrane, driven by soluble N-ethylmaleimide-sensitive factor attachment protein receptor (SNARE) complexes and synaptotagmin family proteins [41–43].

After elucidating the biogenesis of EXOs, research efforts have shifted toward modulating their secretion through preconditioning strategies tailored for MSCs. A central concept underlying these approaches is that MVBs reside at a fate-determining junction: they may undergo fusion with lysosomes, leading to content degradation, or alternatively fuse with the plasma membrane to release EXOs. Thus, strategies that suppress lysosomal activity or promote MVB–plasma membrane fusion can bias MVB trafficking toward the secretory route. For example, under three-dimensional (3D) culture conditions, the downregulation of integrin α1 in MSCs inhibits the Ras homolog gene family member A (RHOA)/cofilin signaling pathway, resulting in the depolymerization of cortical actin [44]. This removal of the cortical actin barrier facilitates MVB docking and fusion with the plasma membrane, ultimately elevating MSC-EXO secretion by ~2.5-fold. Similarly, hypoxic preconditioning upregulates key regulators of EXO biogenesis (*e.g.* ALIX, tumor susceptibility gene 101 [TSG101]) and secretion (*e.g.* RAB27a, RAB27b), thereby enhancing MVB transport and subsequent MVB-plasma membrane fusion [45]. Furthermore, microgravity culture has been established as an effective approach. This method potently promotes MVB-plasma membrane fusion and enhances EV secretion by 7.7-fold. This increase is concomitant with improved immunomodulatory and osteogenic functions, driven primarily by the marked upregulation of RAB27b in MSCs [46]. These molecular changes illustrate how various preconditioning maneuvers collectively direct MVB fate toward EXO release, although the extent and kinetics of this response vary among different MSC sources and preconditioning modalities.

Beyond the regulation of secretion, the biological functions of MSC-EXOs are fundamentally determined by their molecular cargo. These vesicles carry a diverse array of biomolecules, including proteins, lipids, nucleic acids, and metabolites [47]. Proteomic studies have identified hundreds of proteins within MSC-EXOs. These encompass not only evolutionarily conserved exosomal markers and MSC-specific surface antigens, but also a broad spectrum of functional proteins involved in tissue repair and immunomodulation [48, 49]. With respect to nucleic acids, MSC-EXOs are highly enriched in short RNA species, particularly specific microRNAs (miRNAs) [49]. Importantly, the incorporation of miRNAs into EXOs is a highly selective process mediated by the recognition of specific nucleotide sequences, such as EXO-motifs, in coordination with RNA-binding proteins like Aly/REF export factor and fused in sarcoma [50]. This refined cargo-sorting mechanism ensures that MSC-EXOs are equipped with a well-defined molecular repertoire, enabling them to modulate recipient cell functions upon delivery.

#### MVs

In contrast to EXOs, MVs (also referred to as microparticles or ectosomes) are EVs generated through the direct outward budding and fission of the plasma membrane. This mode of biogenesis enables their rapid formation in response to extracellular stimuli or intracellular signals (Table 2) [9, 12,51–53]. Morphologically, MVs are generally larger and more heterogeneous than EXOs, with diameters typically ranging from ~100 to 1000 nm (Figure 1) [12, 54]. They display a buoyant density similar to that of EXOs, generally between 1.13 and 1.19 g·ml^−1^, and are enclosed by a phospholipid bilayer that closely reflects the composition of the parent plasma membrane [55]. In the context of MSCs, MSC-MVs carry characteristic mesenchymal surface markers, such as CD29, CD44, and CD73. These markers not only assist in tracing their cellular origin but may also influence their biodistribution and interactions with target cells [56]. Across multiple cell types and under various stimulatory conditions, MV biogenesis is often accompanied by the loss of membrane lipid asymmetry, most notably the externalization of phosphatidylserine on the outer leaflet [52, 53]. This phosphatidylserine exposure allows for detection *via* Annexin V binding, making it a widely used functional marker in flow cytometry and imaging [28]. It should be noted, however, that not all MV populations exhibit pronounced phosphatidylserine externalization or Annexin V positivity; under certain conditions, MVs may form while preserving lipid asymmetry [57, 58]. Therefore, Annexin V-based assays should be interpreted in conjunction with other characterization approaches, with due consideration paid to the cellular source and physiological context. Collectively, these definitional, biophysical, and surface-marker characteristics establish MVs as a distinct EV subtype. Although considerably heterogeneous, they are readily identifiable and possess significant potential for both mechanistic studies and translational applications in MSC-EV-based tissue regeneration.

Unlike EXOs, which derive from the endosomal pathway, MVs are formed by the direct outward budding and fission of the plasma membrane. This process is typically initiated by cellular activation or stress, which elevates intracellular Ca^2+^ levels and induces membrane lipid reorganization along with the remodeling of the subcortical actin cytoskeleton [51–53, 59]. In mammalian cells, including MSCs, MV biogenesis involves a coordinated sequence of lipid redistribution and cytoskeletal rearrangement. Specifically, the Ca^2+^-dependent activation of aminophospholipid translocases (flippases and floppases), scramblases, and the protease calpain leads to the loss of phospholipid asymmetry, which is marked by phosphatidylserine exposure. Concurrently, calpain causes the disassembly of the actin cortex; together, these actions dramatically promote membrane curvature and budding [60–62]. Simultaneously, the Ras homology (RHO) family of small GTPases and Rho-associated protein kinase (ROCK) stimulate actin dynamics and actomyosin contractility to facilitate vesicle formation. Following this, the small GTPase ADP-ribosylation factor (ARF) 6, often functioning in concert with ARF1, promotes myosin light chain phosphorylation and actomyosin contraction to ultimately drive vesicle scission [63–67]. Although the ESCRT machinery is primarily associated with endosomal sorting, it also contributes to MV formation. For example, the biogenesis of arrestin domain-containing protein 1–mediated MVs requires ESCRT components such as TSG101 and vacuolar protein sorting 4 for successful membrane scission [68]. Thus, MV biogenesis can be summarized as a multi-step process involving Ca^2+^-induced lipid scrambling, Rho/ROCK-dependent actomyosin remodeling, ARF6-guided cargo sorting and contractile activity, calpain-mediated cortical breakdown, and context-dependent ESCRT assistance. This mechanistic framework clearly distinguishes MV formation from the endosomal biogenesis of EXOs.

Building on the mechanistic scheme outlined above, the secretion of MVs is directly regulated by the activation or stress status of the cell. Consequently, it can be modulated through preconditioning strategies that target Ca^2+^-dependent lipid scrambling and actomyosin remodeling. The elevation of intracellular Ca^2+^ by agents such as ionophores, as well as by the stimulation of purinergic P2X7 receptors with adenosine triphosphate (ATP), is a well-recognized stimulus that enhances plasma membrane budding and the subsequent shedding of MVs across various cell types [52, 53, 69, 70]. In addition, our studies have revealed that the hypoxic microenvironment within MSC aggregates activates hypoxia-inducible factor 2-alpha (HIF-2α) signaling, which functions as a key driver enhancing the biogenesis and release of MSC-MVs [71].

Unlike the selective cargo packaging observed in endosomes, the loading of molecular cargo into MVs occurs concomitantly with plasma membrane budding. This process entraps adjacent cytosolic components alongside membrane-associated molecules. As a result, shed MVs typically expose phosphatidylserine and are enriched in lipid raft proteins, although the precise molecular rules governing this cargo selection remain incompletely resolved [72]. Consistent with this mechanism, MVs are capable of transporting diverse bioactive molecules. These include membrane receptors, functional proteins, and nucleic acids that can be translated or exert post-transcriptional regulatory effects in recipient cells [72]. In MSCs specifically, MSC-MVs have been shown to deliver messenger RNA (mRNA) transcripts, such as those encoding small ubiquitin-like modifier 1. This delivery leads to *de novo* protein synthesis, which subsequently enhances cell proliferation, resistance to apoptosis, and distinct mesenchymal phenotypic traits [62]. These functional effects are abolished upon RNA inactivation. Furthermore, the RNA profile of MSC-MVs reveals a distinct subset of transcripts and characteristic miRNA patterns, strongly supporting a regulated, rather than stochastic RNA packaging process [62, 73]. Reflecting their direct biogenesis from the plasma membrane, MSC-MVs can also encapsulate complex cytoplasmic structures, including functional organelles. This highlights their impressive capacity to deliver multifaceted cargo far beyond simple proteins and RNAs [72]. In our recent studies, we further demonstrated that MSC-MVs derived from aggregated MSCs can successfully transfer functional lysosomal and Golgi components to recipient cells, resulting in the restoration of corresponding organellar activities [71, 74].

#### ApoVs

ApoVs constitute a distinct category of EVs generated specifically during the execution phase of programmed cell death, in contrast to EXOs and MVs, which are released from viable cells (Table 2) [75]. Apoptosis involves effector caspase activation, leading to a stereotypic sequence of cellular changes, including cell shrinkage, chromatin condensation, membrane blebbing, and ultimately cellular fragmentation. During this process, portions of the dying cell pinch off, or the entire cell disassembles into membrane-bound vesicles [76, 77]. These vesicles sequester intracellular constituents and expose PS, allowing for their prompt uptake by phagocytes. This rapid clearance ensures an immunologically silent clearance that prevents secondary necrotic inflammation [78]. ApoVs exhibit considerable heterogeneity in both size and cargo composition, with diameters ranging from 50 to 5000 nm and a buoyant density of ~1.16 to 1.28 g·ml^−1^ (Figure 1) [55, 78, 79]. The near-universal externalization of phosphatidylserine renders ApoVs strongly positive for Annexin V binding, a feature that distinguishes them from many MV populations that may maintain lipid asymmetry [80]. Taken together, these morphological and biochemical characteristics establish ApoVs as a highly heterogeneous population of debris-removal vesicles. Their complex molecular content is increasingly recognized as a source of bioactive signals with potential relevance for MSC-EV-based regenerative strategies.

ApoVs are generated specifically during the execution phase of apoptosis following the activation of executioner caspases [80]. Upon the activation of caspase-3/7, the cleavage of ROCK1 occurs. This event, together with the phosphorylation of blebbing regulators such as myosin light chain (MLC) and the caspase-cleaved receptor Plexin-B2 (Plxnb2), induces sustained cortical actomyosin contraction and vigorous membrane blebbing [81]. This process results in the pinching off of heterogeneously sized ApoVs from the retracting plasma membrane. Concurrently, the caspase-dependent activation of the phospholipid scramblase Xk-related protein 8, along with the inactivation of ATP-dependent aminophospholipid flippases, leads to the collapse of bilayer lipid asymmetry. This collapse results in the near-universal externalization of phosphatidylserine on newly formed vesicles [78, 82]. Beyond conventional blebbing, caspase-activated pannexin-1 (PANX1) channels promote the formation of elongated ‘beads-on-a-string’ apoptopodia. Supported by transient apoptotic microtubule spikes, these protrusions undergo serial fragmentation into chains of ApoVs, a process which is further enhanced *in vitro* by fluid shear stress and physical contact with neighboring cells [79, 83, 84]. A complementary endosomal pathway also contributes to biogenesis, wherein caspase-3-mediated sphingosine-1-phosphate (S1P)/S1P receptors (S1PR) signaling activates the actin cytoskeleton and drives the budding of apoptotic EXO-like vesicles from MVBs [85, 86]. In summary, the executioner caspase-ROCK1/MLC/PLXB2 blebbing axis, PANX1/microtubule-assisted protrusion scission, the endosomal S1P/S1PR pathway, and the obligatory externalization of phosphatidylserine collectively represent the defining biogenetic and molecular features that differentiate ApoVs from other EVs.

Operationally, MSC-ApoVs can be reliably generated through specific apoptosis-preconditioning protocols. Commonly employed inductors include staurosporine (STS), ultraviolet (UV) irradiation, nutrient deprivation, and apoptosis-receptor ligation [80, 87, 88]. These stimuli converge mechanistically on caspase activation, initiating the caspase-dependent execution pathways that drive membrane blebbing, apoptopodia formation, and vesicle scission cascades described previously. Notably, apoptosis induction significantly enhances vesicle yield. Compared to baseline release, MSCs undergoing apoptosis produce substantially more ApoVs, with yield increases of up to 10-fold having been observed [89]. Furthermore, under identical conditions, MSCs have been reported to generate larger quantities of ApoVs than certain immune cell types, highlighting the scalability of MSC-ApoV production for translational applications [89]. In terms of cellular identity, MSC-ApoVs retain key mesenchymal surface markers [90]. Proteomic and targeted molecular analyses further confirm that MSC-ApoVs carry a characteristic set of parental cell proteins along with apoptosis-specific components, thereby reinforcing both their traceability and functional relevance [90]. Collectively, these established induction methods, yield advantages, and identity-retention characteristics position MSC-ApoVs as a practically manageable and phenotypically traceable EV subclass well-suited for integration into MSC-EV-based tissue regeneration workflows.

Consistent with their origin in apoptotic cells, ApoVs encapsulate a diverse array of intracellular components, including chromosomal deoxyribonucleic acid (DNA), micronuclei, intact or partially degraded organelles (*e.g.* mitochondria and Golgi fragments), lipids, proteins, and a broad spectrum of RNA species [75,78–80, 91]. This composition enables ApoVs to sequester and transfer complex biomolecular information to recipient cells. Proteomic studies further confirm that MSC-ApoVs retain characteristic mesenchymal markers (*e.g.* CD29, CD44, CD90) as well as apoptosis-associated proteins such as apoptosis-mediating surface antigen FAS (FAS), integrin-α5, syntaxin-4, caveolin-1, caveolae-associated protein 1 (cavin-1), and calreticulin [90]. Collectively, ApoVs convert the inevitability of cell death into a deliverable, immunologically silent, and tunable source of bioactive signals with potential applications in tissue regeneration and therapy. However, several inherent challenges must be addressed before clinical translation can be fully realized. These challenges include heterogeneity among ApoV subtypes, induction-dependent compositional variation, and immense cargo complexity. Additionally, there remains a critical need for standardized isolation methods, precise definitions, and controlled release systems. Addressing these issues will require established frameworks and harmonized methodologies to ensure safety and efficacy.

### Therapeutic applications of MSC-EVs in tissue regeneration

**Figure 2:**
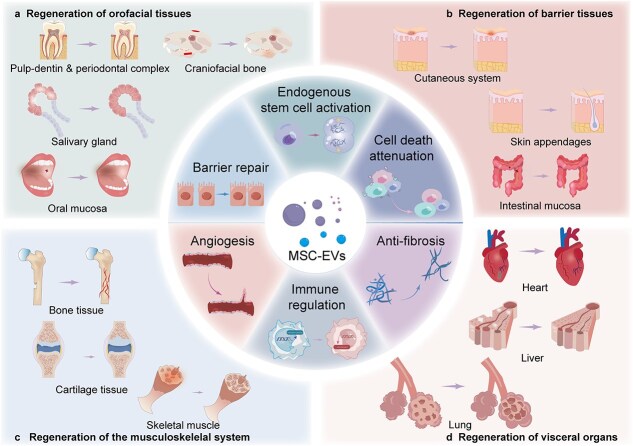
MSC-EVs orchestrate multi-tissue regeneration through shared mechanisms. This schematic illustrates how MSC-EVs orchestrate multi-tissue regeneration through six core mechanisms (inner ring): the restoration of tissue barriers, the activation of endogenous stem cells, the attenuation of cell death, anti-fibrosis, immune regulation, and angiogenesis. These mechanisms collectively enable the regeneration of: (**a**) orofacial tissues, (**b**) barrier tissues, (**c**) the musculoskeletal system, and (**d**) visceral organs. *MSC-EVs* mesenchymal stem cell-derived extracellular vesicles

MSC-EVs have emerged as pivotal mediators of tissue repair and regeneration across diverse organ systems. They orchestrate a conserved set of regenerative mechanisms, including immunomodulation, angiogenesis, anti-fibrosis, the activation of endogenous stem cells, the restoration of barrier integrity, and the regulation of cell death pathways (Figure 2). Through these functions, MSC-EVs effectively remodel pathological microenvironments and promote functional recovery within a cell-free therapeutic framework. Consequently, these shared biological properties establish MSC-EVs as a versatile therapeutic platform with the potential to address a wide spectrum of diseases characterized by inflammation, ischemia, fibrosis, and structural degeneration.

This section provides a systematic review of the therapeutic applications of MSC-EVs in orofacial, barrier, musculoskeletal, and visceral tissue regeneration. We specifically examine their efficacy in treating a range of conditions, such as pulp necrosis, periodontitis, craniofacial bone defects, salivary gland dysfunction, skin wounds, oral and intestinal mucosal injuries, bone and muscle defects, osteoarthritis, and damage to cardiac, hepatic, and pulmonary tissues (Figure 2). Furthermore, we emphasize how engineered MSC-EVs enhance targeting precision, systemic stability, and overall regenerative outcomes. Such engineered vesicles are typically developed through deliberate strategies, including cargo modulation, surface functionalization, or integration with biomaterial carriers. Ultimately, these bioengineering advancements rapidly accelerate the translation of MSC-EV therapies from preclinical validation into localized clinical application.

#### Regeneration of orofacial tissues

##### Regeneration of the pulp-dentin complex

The pulp-dentin complex constitutes an integrated functional unit, consisting of a highly vascularized and innervated pulp core enveloped by a mineralized dentin scaffold. Conventional root canal therapy effectively eliminates infection but results in the irreversible loss of this vital structure [92]. True regeneration therefore necessitates the coordinated reconstitution of both the soft pulp tissue and the dentin layer, the latter of which is maintained by functional odontoblasts. Direct MSC transplantation is hampered by high costs and safety considerations, and chemotactic cell homing remains unpredictable in necrotic root canals. Consequently, MSC-EVs represent a promising cell-free alternative [93]. Given that pulp necrosis is primarily driven by bacterial-induced inflammation and hypoxic damage, MSC-EVs hold particular appeal. They possess a dual capacity to both modulate this pathological microenvironment and promote the integrated regeneration of the pulp-dentin complex (Table 3) [94–112].

**Table 3:** Functional properties of MSC-EVs in orofacial tissue regeneration

EV source	Species	Model	Engineering strategy	Outcomes	Ref.
Swine DPSCs	Nude mice	Ectopic dental pulp regeneration model	——	Pulp-like tissue formation↑ Collagen deposition↑ Vascularization↑	[94]
Human DPSCs	1. Nude mice 2. Beagle dogs	Ectopic/orthotopic dental pulp regeneration model	——	Pulp-like tissue regeneration↑ Vessel density↑ Dentin-like tissue formation↑	[95]
Human DPSCs	Rats	Pulpotomy	Encapsulation in PLGA-PEG-PLGA microspheres	Dentin bridge formation↑ Mineralization↑ Collagen deposition↑ Inflammation↓	[96]
SHED	Immunodeficient mice	Ectopic dental pulp regeneration model	3D aggregate culture	Pulp-like tissue regeneration↑ Angiogenesis↑ Dentin bridge formation↑	[97]
Human DPSCs	Mice	Ligature-induced periodontitis	——	Alveolar bone loss↓ Osteoclast formation↓	[98]
Human PDLSCs	——	——	——	Cementoblast proliferation↑ Cementoblast migration↑ Cementoblast mineralization↑ Cementogenic gene expression↑	[99]
SHED	Rats	Alveolar bone defect	Encapsulation in β-TCP scaffold	Bone regeneration↑ Neovascularization↑	[100]
Human DPSCs	Mice	Ligature-induced periodontitis	3D cell culture	Alveolar bone loss↓ Th17 cells in gingiva↓ Treg cells in gingiva↑	[101]
Human BMMSCs	Rats	BRONJ model (tooth extraction)	——	Bone regeneration↑ Angiogenesis↑ Wound healing↑ Senescent cells↓	[102]
Human DPSCs	Rats	Mandibular bone defect	Laden with collagen membrane	New bone density↑ Wound closure↑ Osteogenic gene expression↑	[103]
Rat BMMSCs	Rats	1. Senescent mandibular bone fracture 2. Senescent mandibular critical-sized defect	1. Membrane modification with Apt19s (DNA aptamer) 2. Loaded with miR-376b-5p 3. Encapsulated in GelMA hydrogel	Endogenous stem cell recruitment↑ New bone formation↑ Cellular senescence↓	[104]
Human BMMSCs	Mice	Calvarial defect	Encapsulation in decellularized fish scale scaffold	New bone formation↑ Bone volume/Tissue volume↑ Trabecular number↑	[105]
Human tonsil MSCs	Rats	Ovariectomy-induced submandibular gland dysfunction	——	Submandibular gland dysfunction↓ AQP5 expression↑ α-amylase expression↑	[106]
Human DPSCs	Mice	Sjögren's syndrome	——	Salivary flow rate↑ AQP5 expression↑ Autoantibody levels↓	[107]
Rat BMMSCs	Rats	Streptozotocin-induced diabetic salivary gland dysfunction	——	Gland structure improvement↑ Serum amylase↓ Salivary IgA↓	[108]
Rat BMMSCs	Rats	Cisplatin-induced parotid salivary gland damage	——	Vacuolation↓ Apoptotic cells↓ Oxidative stress↓	[109]
Human ADMSCs	Mice	Arecoline-induced OSF	——	Pathological damage of OSF↓ Submucosal collagen deposition↓ Autophagy levels in myofibroblasts↓	[110]
Mouse GMSCs	Mice	Gingival full-thickness wound	TNF-α pretreatment	Gingival wound healing↑ Local gingival inflammation↓	[111]
Human GMSCs	Rats	Critical-sized tongue defect	Laden with SIS-ECM scaffold	Taste bud regeneration↑ Re-innervation of taste buds↑ Lingual papillae regeneration↑ Epithelial recovery↑	[112]

*MSC* mesenchymal stem cell, *EV* extracellular vesicle, *DPSCs* dental pulp stem cells, *PLGA-PEG-PLGA* poly(lactic-co-glycolic acid)-poly(ethylene glycol)-poly(lactic-co-glycolic acid), *SHED* stem cells from human exfoliated deciduous teeth, *3D* three-dimensional, *PDLSCs* periodontal ligament stem cells, *β-TCP* β-tricalcium phosphate, *BMMSCs* bone marrow mesenchymal stem cells, *Th17 cells* T helper 17 cells, *Treg cells* regulatory T cells, *BRONJ* bisphosphonate-related osteonecrosis of the jaw, *GelMA* gelatin methacryloyl, *AQP5* aquaporin 5, *IgA* immunoglobulin A, *ADMSC* adipose-derived mesenchymal stem cells, *OSF* oral submucosal fibrosis, *GMSC* gingiva-derived mesenchymal stem cells, *TNF-α* tumor necrosis factor-alpha, *SIS-ECM* small intestinal submucosa-extracellular matrix

Mechanistically, MSC-EVs coordinate pulp regeneration through three principal pathways. First, they promote odontoblastic differentiation and dentin mineralization. They achieve this by upregulating key dentinogenic markers, such as dentin sialophosphoprotein, dentin matrix protein-1, alkaline phosphatase, and runt-related transcription factor 2. This upregulation is driven heavily by the activation of the transforming growth factor (TGF)-beta (β)/small mother against decapentaplegic (Smad) signaling axis [94,113–115]. Second, MSC-EVs exert pro-angiogenic effects through multiple mechanisms. For instance, EVs derived from aggregated stem cells from human exfoliated deciduous teeth (SHED-EVs), which are enriched in miRNA (miR)-26a, significantly enhance angiogenesis and pulp regeneration *in vivo* by activating the TGF-β/Smad2/3 pathway [97]. Notably, recent studies have revealed that within the ischemic and hypoxic microenvironment of the root canal, transplanted dental pulp MSCs (DPSCs) undergo apoptosis. The resulting ApoVs are subsequently internalized by endothelial cells and deliver the mitochondrial protein Tu translation elongation factor, mitochondrial, thereby activating the transcription factor EB (TFEB)-mediated autophagy pathway. This process potently enhances the angiogenic capacity of endogenous endothelial cells and has enabled the regeneration of vascularized pulp-like tissue in a large animal model [95]. Third, although less extensively studied, MSC-EVs showcase considerable potential in supporting neuroregeneration. Gingival MSC (GMSC)-EVs, for example, enhance Schwann cell proliferation and migration by modulating c-JUN/Notch1 repair phenotypes and upregulating neurogenic markers in dental papilla cells [116]. Furthermore, it is noteworthy that revascularization and reinnervation often occur concurrently within these regenerated pulp-like tissues.

To enhance the translational potential of MSC-EVs, researchers have developed formulations involving their encapsulation within injectable hydrogels, such as peptide-modified or thermosensitive chitin-based matrices. This engineering strategy protects MSC-EVs from rapid clearance, ensures conformal adaptation to irregular root canal anatomy, and enables a sustained release profile. *In vivo* studies demonstrate that such systems facilitate the regeneration of pulp-like tissue characterized by aligned collagen deposition, polarized odontoblast layers, and integrated vascular and neural networks [94, 117, 118]. Similarly, controlled-release systems employing polyethylene glycol-poly lactic acid-co-glycolic acid (PEG-PLGA) microspheres or amphiphilic triblock copolymers prevent an initial burst release, ultimately supporting the formation of structured dentin bridges [96]. Beyond conventional delivery platforms, engineered EVs can be loaded with lineage-specifying regulators, such as nuclear factor I/C, to directly counteract inflammation-mediated inhibition and enhance the dentinogenic capacity of apical papilla-derived MSCs in models of apical periodontitis [119]. Furthermore, combining decellularized tooth matrix (DTM) with DPSC aggregates has been shown to reestablish an odontogenic niche that amplifies EV secretion [120]. These locally secreted EVs subsequently promote odontogenic and angiogenic differentiation, leading to functional tooth regeneration observed in both preclinical and clinical studies.

##### Regeneration of the periodontal complex

The periodontal complex is an integrated functional unit comprising the gingiva, periodontal ligament, cementum, and alveolar bone. Together, these components secure teeth within the jaw and maintain oral tissue homeostasis. Following a periodontal injury, dysbiotic biofilms activate Toll-like receptor 4 (TLR4)/nuclear factor κB (NF-κB) signaling, which leads to the increased production of TNF-α, interleukin (IL)-1, IL-6, and IL-8. These cytokines enhance matrix metalloproteinase (MMP) activity, promote receptor activator of NF-κB ligand–mediated osteoclastogenesis, and drive the progressive destruction of the constituent tissues [121]. The concurrent infiltration of neutrophils and lymphocytes, along with microvascular remodeling, sustains a state of oxidative stress that perpetuates tissue breakdown [121]. Although scaling and root planing combined with guided tissue regeneration remain the standard treatments, their capacity to predictably restore lost tissues remains limited [122]. Furthermore, the transplantation of MSCs faces challenges related to safety and high costs. In this context, MSC-EVs, which efficiently deliver anti-inflammatory, osteogenic, cementogenic, and pro-angiogenic factors, represent a promising cell-free strategy for achieving functional periodontal complex regeneration (Table 3).

During the inflammatory phase of periodontal healing, MSC-EVs derived from the periodontal ligament, gingiva, dental pulp, or exfoliated deciduous teeth attenuate NF-κB signaling, lower the levels of IL-1β and TNF-α, and promote immunomodulatory responses by polarizing macrophages toward an anti-inflammatory M2 phenotype and enhancing regulatory T (Treg) cell activity [123]. Notably, MSC-ApoVs exhibit a heightened immunoregulatory capacity. Recent evidence indicates that BMMSC-ApoVs are internalized by macrophages and mitigate lipopolysaccharide (LPS)-induced inflammation *via* the AMP-activated protein kinase (AMPK)/Sirtuin 1 (SIRT1)/NF-κB axis. This molecular interaction leads to a dose-dependent reduction in TNF-α and IL-6 alongside increased IL-10 secretion [124]. Additionally, these ApoVs suppress osteoclast differentiation and bone resorption by downregulating the release of TNF-α from pro-inflammatory macrophages, thereby contributing to the preservation of periodontal bone [124]. In a complementary manner, conventional MSC-EVs also inhibit osteoclastogenesis. This helps to reestablish osteo-immunological equilibrium and further limits inflammatory bone loss [98].

During the reparative phase of periodontal healing, MSC-EVs enhance the proliferation and migration of periodontal ligament cells through the CD73-mediated activation of the AKT/extracellular signal-regulated kinase (ERK) pathway [125]. Concurrently, they stimulate new bone formation by activating both the Wingless/Integrated (Wnt)/β-catenin and AMPK signaling cascades [100, 126]. A recent paradigm established by Huang *et al.* demonstrates that EVs released from SHED in aggregate cultures exhibit potent odontogenic properties. Enriched with developmental regulators such as axis inhibition protein 2 (Axin2), bone morphogenetic protein (BMP) 2/4, and sonic hedgehog (SHH), these EVs are essential for mobilizing recipient Gli1^+^ stem cells. They enhance the migration, proliferation, and osteogenic or cementogenic differentiation of Gli1^+^ cells, thereby orchestrating a donor-recipient stem cell crosstalk that synergistically regenerates the tooth-periodontal ligament-bone interface and supports the reattachment of avulsed teeth [127]. Furthermore, MSC-EVs enhance the proliferation, migration, and cementogenic mineralization of cementoblasts by activating the phosphatidylinositol 3-kinase (PI3K)/AKT signaling pathway, providing a promising strategy for promoting cementum repair [99].

To further enhance the therapeutic efficacy of MSC-EVs, the preconditioning of MSCs has been employed as a strategic approach. For instance, GMSCs stimulated with TNF-α or DPSCs pretreated with LPS yield EVs with an enriched molecular cargo. These specific EVs promote macrophage polarization toward the M2 phenotype, enhance antioxidant responses, and improve periodontal regeneration *in vivo* [128, 129]. Similarly, cultivating DPSCs under 3D or hypoxic conditions increases both the total EV yield and their pro-angiogenic and anti-inflammatory potency, leading to improved outcomes in periodontitis models [101, 130]. Importantly, the integration of MSC-EVs with biomaterial carriers, such as collagen sponges and β-tricalcium phosphate, enables localized retention and controlled release. Such delivery systems enhance the regeneration of the periodontal ligament, alveolar bone, and vascular networks in both defect and periodontitis models [100, 125].

##### Repair of the craniofacial bone defect

Craniofacial bone defects resulting from trauma, tumor resection, or surgery progress through an initial inflammatory phase, followed by tightly coupled angiogenesis and osteogenesis, and ultimately late remodeling [131, 132]. However, in this anatomically exposed region, persistent microbial challenges, limited perfusion, and local hypoxia frequently derail orderly healing. These factors can even predispose the tissue to osteonecrosis [131–133]. Consequently, regeneration in the skull and jaws hinges on timely immunoregulation and revascularization, which are often inadequate in large defects. In this context, MSC-EVs have gained prominence as cell-free, low-immunogenicity effectors. They concurrently temper inflammation, stimulate neovascularization, and drive osteogenic programs. By doing so, they directly target and resolve the principal bottlenecks associated with cranial and maxillofacial repair (Table 3).

Mechanistically, MSC-EVs intervene during the inflammatory phase of craniofacial bone healing to promote the resolution of immune responses. For example, evidence from a rat model of bisphosphonate-related osteonecrosis of the jaw demonstrates that they suppress senescence and inflammation *via* downregulation of cyclin-dependent kinase inhibitor 1 (P21), retinoblastoma protein, and pro-inflammatory factors. Concurrently, they upregulate the stem cell self-renewal genes *Bmi1* and *Hmg2* [102]. This orchestrated action fosters a pro-regenerative microenvironment that is conducive to angiogenesis and osteogenesis, steering the tissue away from inflammation and toward functional healing. During the subsequent pro-regenerative window, MSC-EVs enhance endothelial angiogenesis. This fundamentally supports the generation of highly vascularized bone within critical-size calvarial defects [134]. Thereafter, DPSC-EVs augment jawbone mineral density and induce new bone formation in mandibular defects [103]. Similarly, BMMSC-ApoVs are internalized by recipient BMSCs, thereby boosting their proliferation, migration, and osteogenic differentiation *via* the reactive oxygen species (ROS)/c-Jun N-terminal kinase (JNK) signaling pathway. This sequence of signaling events enhances craniofacial bone repair [135]. Thus, across multiple distinct healing phases, MSC-EVs help to re-balance the immune microenvironment, couple nascent vessels to progenitor osteoblast activity, and sustain the transition from inflammation to structural reconstruction in craniofacial bone.

To enhance the regenerative functions of MSC-EVs *in vivo*, researchers have developed engineered delivery systems and composite biomaterials designed to leverage their inherent therapeutic potential. This principle is exemplified by dual-functionalized MSC-EVs that are engineered with a stem cell-homing Apt19s (DNA aptamer) on the membrane and osteogenic miR-376b-5p within the lumen. When applied locally, these specially modified MSC-EVs sequentially recruit endogenous stem cells and promoted bone formation in mandibular defects [104]. Similarly, EVs derived from neural EGFL-like 1 (Nell1)-overexpressing MSCs exhibit an enhanced osteogenic capacity. This stems directly from a targeted downregulation of miR-25-5p and the subsequent activation of the Smad2/ERK pathway. These tailored vesicles have shown immense promise in boosting acellular bone regeneration in calvarial defects [136]. Further extending this strategy, a decellularized fish scale scaffold has been utilized to load and deliver BMMSC-EVs. Its inherent hierarchical porosity enables high EV-loading capacity and sustained release, while its native collagenous composition ensures excellent biocompatibility. This MSC-EV-enriched scaffold significantly enhanced calvarial bone regeneration in mice, underscoring the value of biomimetic, natural biomaterial-based EV delivery platforms [105].

##### Regeneration of the salivary gland

Salivary glands are vulnerable to a spectrum of clinical insults. These include head and neck irradiation, which induces cellular senescence and oxidative stress, and chemotherapy, which triggers apoptosis and ROS generation. Additional insults include hyperglycemia-driven structural remodeling, menopause-related xerostomia, and autoimmune Sjögren’s syndrome, which is characterized by ductal inflammation. Furthermore, aging-associated ‘inflammaging’ often leads to fibrosis and the loss of aquaporin 5 (AQP5) expression. Despite their diverse origins, these pathological processes ultimately converge to disrupt secretory function and diminish salivary flow. In this context, MSC-EVs have emerged as promising cell-free therapeutic agents. They combine practical advantages for salivary gland repair with a growing level of translational relevance (Table 3).

Despite promising preliminary results, the peer-reviewed literature that systematically evaluates MSC-EVs for salivary gland regeneration remains limited. This highlights a clear need for broader investigations across diverse models. The therapeutic effects of MSC-EVs are multifaceted. During inflammatory and autoimmune phases, they suppress pro-inflammatory mediators and recalibrate adaptive immunity. This is evidenced by reduced TNF-α and IL-6 levels in ovariectomy models. Additionally, these vesicles help expand Treg populations and restrain T helper (Th) 17 cell responses, leading to cytokine profile shifts in Sjögren’s syndrome models [106, 107, 137]. In the context of fibrotic remodeling, MSC-EVs inhibit TGF-β/Smad signaling and downregulate collagen I/III and TGF-β1 expression, correlating with the histological amelioration of fibrosis [106, 108]. Under cytotoxic and oxidative stress conditions, they alleviate apoptosis and oxidative damage, counteracting cellular senescence through downregulation of senescence-associated beta-galactosidase (SA-β-gal) and enhancement of superoxide dismutase (SOD) activity [109, 138]. Critically, MSC-EVs also restore secretory function by upregulating AQP5 expression in acinar cells and enhancing pilocarpine-stimulated salivary flow in autoimmune settings [106, 107,137–139]. Together, these coordinated mechanisms enable MSC-EVs to facilitate salivary gland regeneration across diverse injury types by targeting convergent pathways involving inflammation, fibrosis, oxidative stress, and secretory dysfunction.

Building upon these multifaceted repair mechanisms, the therapeutic potential of EVs for salivary gland regeneration can be significantly enhanced. This enhancement is achieved through the precision screening of potent subpopulations and the engineered cultivation of EV-secreting cells. It is noteworthy, however, that current research on engineered vesicles or EV-composite materials in this field remains limited regarding the use of MSC-EVs. For instance, Park *et al.* identified that CD9^+^ EVs derived from salivary gland basal progenitor cells—not MSCs—are enriched with miR-3162 and miR-1290. These miRNAs downregulate activin receptor type I and inhibit Smad2/3 phosphorylation, thereby exerting anti-fibrotic effects [140]. In a complementary engineering approach, Cho *et al.* utilized a Wnt3A-releasing microwell scaffold to culture salivary gland epithelial stem cells, which markedly increased EV production and reinforced their regenerative capacity *via* the 14–3-3 protein zeta/delta (YWHAZ)/PI3K/AKT pathway [141]. To improve delivery, EV-hydrogel composites enable localized sustained release. When combined with retroductal administration, these composites can maximize EV retention and bioavailability at the target site. Together, the integration of precision EV isolation, engineered cell culture systems, and advanced delivery platforms will accelerate the translation of EV-based therapies toward clinical salivary gland regeneration.

##### Regeneration of the oral mucosal tissues

The oral mucosa exhibits a remarkable capacity for regeneration, healing more rapidly and with significantly less scarring than skin [142]. This superior healing phenotype is attributed to the fetal-like characteristics of oral mucosal fibroblasts, which resemble the fibroblasts responsible for scarless wound healing in fetal skin [142]. However, this inherent regenerative potential can be disrupted by pathological conditions, such as oral submucous fibrosis (OSF). In OSF, stimuli like arecoline drive the transformation of fibroblasts into myofibroblasts. This cellular transformation leads to excessive collagen deposition and tissue fibrosis [110]. Similarly, orofacial soft tissue defects resulting from surgical trauma, radiotherapy, or congenital anomalies like cleft lip and palate can trigger aberrant healing responses. These responses result in scarring and fibrosis that impair maxillofacial growth, speech, and mastication [143]. Conventional therapies for these conditions, such as surgery or drug injections for OSF, often only alleviate symptoms without reversing the underlying pathology [144]. In this context, MSC-EVs have emerged as a promising therapeutic modality. They recapitulate the paracrine immunomodulatory and anti-fibrotic actions of their parent cells, making them rational candidates for promoting functional oral mucosal regeneration (Table 3).

MSC-EVs play a pivotal role in orchestrating the regeneration of oral mucosal tissues by modulating key cellular processes. For instance, in the context of OSF, ADMSC-EVs deliver miR-125a-5p, which directly suppresses the TGF-β/Smad2 axis [110]. This targeted molecular intervention effectively inhibits the transformation of resident fibroblasts into collagen-producing myofibroblasts, thereby attenuating the excessive collagen deposition and tissue stiffness characteristic of OSF. Beyond their anti-fibrotic role, MSC-EVs significantly enhance the regenerative phase of healing. Kou *et al.* demonstrated that GMSCs possess an intrinsically superior capacity to produce and secrete IL-1RA-packed EVs compared to skin MSCs [111]. This specialized secretion process is governed by a unique Fas/Fas-associated phosphatase 1 (Fap-1)/caveolin-1 molecular axis that regulates SNARE-mediated exocytosis. Functional *in vivo* studies confirmed that GMSC-EVs accelerate palatal wound healing in an IL-1RA-dependent manner. Notably, this regenerative effect proved to be far more potent than the healing induced by skin MSC-EVs applied to dermal wounds. This multifaceted approach, which tackles the underlying fibrotic pathology while promoting tissue restitution, underscores the immense therapeutic potential of native MSC-EVs in restoring functional oral mucosa.

The inherent regenerative capabilities of MSC-EVs can be further augmented through engineering strategies designed to enhance their targeting and efficacy for specific oral mucosal conditions. For example, priming GMSCs with the pro-inflammatory cytokine TNF-α results in the production of EVs with an enriched anti-inflammatory profile, which includes elevated levels of the ectoenzyme CD73 [145]. Alternatively, biomaterial-based strategies focus on achieving sustained local delivery. Incorporating MSC-EVs into biocompatible scaffolds, such as porcine small intestinal submucosa-extracellular matrix (ECM) constructs, creates a protective reservoir that facilitates the gradual release of EVs at the defect site [112]. This engineered method has proven effective in complex regeneration scenarios, such as the healing of myomucosal tongue defects, where it supported the restoration of specialized structures, including both papillae and taste buds.

#### Regeneration of barrier tissues

##### Cutaneous wound repair

Normal cutaneous wound repair proceeds through sequential phases of hemostasis, inflammation, proliferation, and remodeling. In contrast, chronic and diabetic wounds frequently arrest in a persistent inflammatory state. This state is characterized by tissue hypoxia, oxidative stress, impaired angiogenesis, and delayed re-epithelialization and collagen deposition [146]. Although advanced wound management has been improved by techniques such as topical growth factors, ECM-based products, and bioengineered skin substitutes, complete closure is still not achieved in up to half of all cases [147]. MSC-EVs offer a promising alternative by recapitulating the multifaceted paracrine functions of MSCs across these wound healing stages [146, 147]. They modulate inflammatory responses, promote angiogenesis, stimulate keratinocyte and fibroblast proliferation, and support structured remodeling. All of these functions occur within a cell-free framework that confers favorable safety and low immunogenicity (Table 4) [148–157].

**Table 4:** Functional properties of MSC-EVs in barrier tissue regeneration

EV source	Species	Model	Engineering strategy	Outcomes	Ref.
Human UCMSCs	Rats	Skin deep second-degree burn model	——	Wound healing↑ Angiogenesis↑	[148]
Human UCMSCs	Rats	Skin deep second-degree burn model	——	Re-epithelialization↑ Collagen I/III ratio↑ Skin cell apoptosis↓	[149]
Human ADMSCs	Rats	Diabetic foot ulcer	Nrf2 overexpression	Wound closure↑ Granulation tissue formation↑ Angiogenesis↑	[150]
Human ADMSCs	Rats	Diabetic wound	1. Hypoxic pretreatment 2. Circ-Snhg11 overexpression	Wound healing↑ Angiogenesis↑ Inflammation↓ Endothelial progenitor cell apoptosis↓	[151]
Mouse BMMSCs	Mice	Depilation-induced telogen-to-anagen transition	——	Telogen-to-anagen transition↑ Hair regrowth↑ Hair follicle activation↑	[152]
Mouse ADMSCs	Nude mice	*De novo* hair follicle regeneration	——	Hair number↑ Hair follicle maturation↑ Anagen-associated growth factors↑ Catagen-associated factors↓	[153]
Human UCMSCs	Mice	DSS-induced colitis; TNBS-induced colitis	——	Mucosal healing↑ Intestinal epithelial integrity↑ Lgr5^+^ ISC regeneration↑ Epithelial proliferation↑	[154]
Human UCMSCs	Mice	DSS-induced colitis	——	Colonic inflammation↓ Intestinal tissue damage↓	[155]
Human UCMSCs	Rats	DSS-induced colitis	1. IL-27 overexpression 2. Encapsulation into bioadhesive GelMA/DMA hydrogel microcarriers	Colonic inflammation↓ Intestinal epithelial barrier integrity↑ Tight junction proteins↑	[156]
Rat BMMSCs	Rats	DSS-induced colitis	EphB2 overexpression	Colonic inflammation↓ Intestinal barrier integrity↑ Oxidative stress↓	[157]

*MSC* mesenchymal stem cell, *EV* extracellular vesicle, *UCMSCs* umbilical cord mesenchymal stem cells, *ADMSCs* adipose-derived mesenchymal stem cells, *Nrf2* nuclear factor erythroid 2-related factor 2, *Circ-Snhg11* circular RNA Snhg11, *BMMSCs* bone marrow mesenchymal stem cells, *DSS* dextran sulfate sodium, *TNBS* 2,4,6-trinitrobenzenesulfonic acid, *Lgr5* leucine-rich repeat-containing G-protein coupled receptor 5, *ISC* intestinal stem cell, *IL-27* interleukin-27, *GelMA* gelatin methacryloyl, *DMA* dopamine methacrylamide, *EphB2* ephrin type B receptor 2

During the early inflammatory phase, UCMSC-EVs enriched in miR-181c suppress TLR4 signaling, thereby mitigating hyperinflammation following burn injury. In parallel, LPS-primed UCMSC-EVs, which are highly enriched in the miRNA let-7b, promote macrophage polarization toward the M2 phenotype and reduce the secretion of pro-inflammatory cytokines [158, 159]. Similarly, MSC-ApoVs have been demonstrated to enhance M2 macrophage polarization and inhibit pyroptosis by attenuating oxidative stress, accelerating wound closure in both normal and diabetic mouse models [160, 161]. As healing progresses to the angiogenic stage, pro-vascular cargoes delivered by MSC-EVs play a decisive role. For instance, Wnt4-positive UCMSC-EVs activate β-catenin signaling in endothelial cells [148]. Concurrently, nuclear factor erythroid 2-related factor 2 (Nrf2)-overexpressing ADMSC-EVs upregulate senescence marker protein-30 (SMP30), vascular endothelial growth factor (VEGF), and vascular endothelial growth factor receptor 2 (VEGFR2) phosphorylation, thereby promoting endothelial progenitor cell proliferation and new vessel formation [150]. Subsequently, MSC-EVs support the proliferative phase of repair. Both BMMSC-EVs and UCMSC-EVs enhance fibroblast and keratinocyte proliferation in a dose-dependent manner. This is accompanied by the upregulation of proliferating cell nuclear antigen (PCNA), cytokeratin-19, and collagen I expression, which facilitates re-epithelialization and granulation tissue formation [148, 149, 162]. Finally, during the tissue remodeling phase, MSC-EVs reduce scarring by modulating key molecular ratios (*e.g.* collagen III:collagen I, TGF-β3:TGF-β1) and inhibiting myofibroblast differentiation. These effects are mediated at least in part by specific miRNAs in MSC-EVs, such as miR-21-5p and miR-125b-5p [163, 164].

To enhance these therapeutic effects, studies demonstrate that stem cell aggregation, hypoxic or inflammatory preconditioning, Nrf2 transfection, or intentional loading of specific molecules such as miR-21-5p or circular RNAs can significantly boost the anti-inflammatory and pro-angiogenic capacity of EVs [150, 151,165–167]. Furthermore, injectable delivery systems, including Pluronic F-127 (PF-127) hydrogel and chitosan-based hydrogels, prolong EV retention to enable sustained release. These systems markedly accelerate wound closure and enhancing vascularization in diabetic ulcer models [168, 169]. Nevertheless, while initial clinical trials confirm the therapeutic potential of MSC-EVs for wound healing, the field now requires large-scale, rigorously controlled trials to establish standardized protocols, determine optimal dosing, and confirm long-term benefits for broad clinical adoption [170].

##### Regeneration of skin appendages

The restoration of fully functional skin requires not only re-epithelialization but also the regeneration of cutaneous appendages. Structures such as hair follicles, sebaceous glands, eccrine sweat glands, and peripheral nerves are essential for reestablishing barrier integrity, thermoregulation, lubrication, and sensory perception [171, 172]. However, current tissue-engineered skin substitutes primarily recapitulate only the epidermal or dermal components, frequently lacking these critical appendages and neural elements. Consequently, patients often experience persistent sensory deficits and chronic pain, highlighting a key limitation in current regenerative strategies [172].

Current research in skin regeneration predominantly focuses on cell-based and scaffold-based approaches. Meanwhile, MSC-EVs remain underexplored in systematic studies. Existing investigations are largely confined to hair follicle biology, thereby overlooking the broader potential of these EVs in multi-appendage regeneration (Table 4). Mechanistically, MSC-EVs coordinate hair follicle regeneration through multiple pathways. They deliver Wnt ligands to activate Wnt/β-catenin signaling, accelerate the telogen-to-anagen transition, and promote a pro-angiogenic microenvironment. In murine models, the intradermal administration of MSC-EVs upregulates cutaneous Wnt3a and Wnt5a. This treatment also increases the expression of the anagen-associated proteoglycan versican in dermal papilla cells, successfully inducing the follicle transition from telogen to anagen phase [152]. Beyond Wnt-mediated signaling, MSC-EVs promote dermal papilla cell proliferation and migration, elevate the levels of VEGF and insulin-like growth factor 1, and facilitate anagen entry following intradermal delivery [152]. In a complementary approach, ADMSC-EVs co-transplanted with dermal and epidermal cells generate more mature follicles *in vivo.* This regeneration is accompanied by the upregulation of platelet-derived growth factor and VEGF, alongside the downregulation of TGF-β1 in the skin tissue [153]. Collectively, these findings establish MSC-EVs as a cell-free platform capable of activating epithelial-mesenchymal signaling pathways and reinstating the anagen program during hair follicle regeneration.

Looking forward, skin appendage stem/progenitor cells may serve as a highly specific source of EVs for appendage regeneration. These cells represent unique epithelial populations residing in the bulge, junctional zone, and duct regions of the tissue. They actively maintain and regenerate appendages, despite their epithelial rather than mesenchymal origin. Importantly, these cells can be directed toward appendage-specific fates both *in vitro* and *in vivo*. For instance, under specific signaling cues, epidermal or epithelial progenitors can be induced to differentiate into sweat gland-like cells and form ductal/secretory structures in engineered skin. Such critical cues include the activation of the epidermal growth factor (EGF)/ERK and ectodysplasin (EDA)/NF-κB pathways [173–175]. This functional plasticity supports the rationale that EVs derived from these progenitor cells may carry biologically relevant signals conducive to appendage regeneration.

##### Regeneration after intestinal injuries

In a range of conditions, spanning from inflammatory and ischemic injuries to neoplastic and iatrogenic damage, the regeneration of the intestinal mucosa is critically impaired by coexisting epithelial barrier defects and sustained inflammatory signaling [176]. Barrier dysfunction presents as the disruption of tight junctions, elevated intestinal epithelial cell death, and dense immune infiltration [177, 178]. Concurrently, ongoing inflammation disrupts the normal coordination of tissue repair mechanisms. Under physiological conditions, repair is initiated by the activation of intestinal stem cells (ISCs), particularly the leucine-rich-repeat-containing G-protein-coupled receptor 5 (Lgr5)-positive population residing at the crypt base [176]. These activated ISCs give rise to a population of proliferative progenitor cells, which then undergo a well-orchestrated sequence of proliferation, differentiation, and migration to replenish the various epithelial cell lineages and restore the mucosal barrier [176]. Under pathological conditions, however, sustained exposure to pro-inflammatory factors and the coordinated induction of diverse cell death pathways (*e.g.* apoptosis, necroptosis, pyroptosis, and ferroptosis), collectively sustain barrier loss and impede mucosal healing [177]. These limitations have prompted the exploration of MSC-EVs as a novel therapeutic approach. These EVs exhibit immunomodulatory and pro-regenerative functions, coupled with low immunogenicity and favorable delivery properties, making them a promising strategy for restoring mucosal homeostasis (Table 4).

In response to the pathological obstacles outlined above, MSC-EVs first recalibrate the inflammatory milieu by decreasing TNF-α, IL-1β, IL-6 while increasing IL-10 and TGF-β, thereby removing cytokine-driven impediments to tissue restitution and restoring mucosal homeostasis [179]. In parallel, UCMSC-EVs accelerate the regenerative axis by promoting ISC activation and epithelial renewal through Wnt pathway engagement [154]. At the barrier interface, MSC-EVs reinforce epithelial tight junctions by upregulating claudin-1 and zonula occludens-1. Moreover, they activate the Snail/Claudins pathway, which further expedites epithelial repair [180, 181]. In addition, BMMSC-EVs significantly attenuate the apoptosis of colonic epithelial cells by suppressing the cleavage of caspase-3, caspase-8, and caspase-9. Concurrently, they alleviate oxidative stress by reducing myeloperoxidase activity and malondialdehyde (MDA) levels while increasing SOD activity and glutathione (GSH) levels [182]. Finally, to counter inflammasome-driven barrier injury, UCMSC-EVs enriched with miR-378a-5p protect against dextran sulfate sodium (DSS)-induced colitis. They achieve this protection through the inhibition of NACHT, LRR, and PYD domains-containing protein 3 (NLRP3) inflammasome activation and subsequent pyroptosis [155].

To enhance the therapeutic potential of MSC-EVs for intestinal repair, engineering strategies focus on improving vesicle targeting and localized efficacy. Nie *et al.* developed MSC-EVs engineered to overexpress the anti-inflammatory cytokine IL-27 [156]. These EVs were then encapsulated into adhesive hydrogel microparticles, creating a system that firmly adheres to the colon for sustained local release, significantly reducing inflammation and promoting barrier healing. In another approach, Yu *et al.* enhanced the inherent homing ability of MSC-EVs by engineering parent MSCs to overexpress ephrin type B receptor 2 (EphB2) [157]. The resulting EphB2-EVs demonstrated superior targeting to inflamed colon tissue following systemic administration. Once localized, they effectively restored immune balance and promoted mucosal repair.

#### Regeneration of the musculoskeletal system

##### Repair of bone defect

The successful repair of bone defects requires the coordinated recruitment and osteogenic differentiation of mesenchymal progenitors, coupled with early and sustained neovascularization to deliver oxygen, nutrients, and signaling molecules to the repair site [183, 184]. The coupling of angiogenesis and osteogenesis is particularly critical in bone tissue and serves as a fundamental principle for effective bone regeneration [183, 184]. Conventional bone tissue engineering strategies rely on three core components: scaffolds, seed cells, and osteoinductive signals. However, their efficacy is often compromised by poor vascular infiltration into the construct and the unstable spatiotemporal presentation of bioactive factors [185]. These limitations frequently result in incomplete or failed healing. In this context, MSC-EVs represent a cell-free therapeutic strategy that intrinsically packages proteins, miRNAs, and other regulatory molecules. This natural packaging enables the targeted delivery of both pro-angiogenic and pro-osteogenic signals to the defect site, thereby effectively coupling angiogenesis with osteogenesis (Table 5) [186–196].

**Table 5:** Functional properties of MSC-EVs in the musculoskeletal system regeneration

EV source	Species	Model	Engineering strategy	Outcomes	Ref.
Mouse BMMSCs	Mice	Femoral fracture	——	Fracture healing↑ Bone and cartilage tissue formation↑Osteoblast differentiation↑	[20]
Mouse BMMSCs	Mice	OVX-induced osteoporosis	——	Bone mass and strength↑ Trabecular bone loss↓ Cartilage damage↓	[186]
Mouse BMMSCs	Mice	OVX-induced osteoporosis	1. Surface modification with bone-targeting peptide (DSS)_6_ 2. Intraluminal loading of RNF146	Bone mass↑ Osteoporosis progression↓	[187]
Human DPSCs	Rats	1. Subcutaneous ectopic osteogenesis 2. Rat femoral condyle defect	1. Osteogenic pre-conditioning 2. Encapsulation into mHA/PEG hydrogel	Bone formation↑ Angiogenesis↑ Inflammation↓	[188]
Mouse BMMSCs	Mice	Cardiotoxin-induced tibialis anterior injury	——	Muscle regeneration↑ Myofiber formation & maturation↑	[189]
Mouse BMMSCs	Mice	Contusion-induced tibialis anterior injury	——	Muscle regeneration↑ Inflammation↓ Fibrosis↓	[190]
Human BMMSCs	Mice	Duchenne muscular dystrophy	Surface functionalization with a dimerized IL6ST decoy receptor	Muscle inflammation↓ Muscle regenerative potential↑	[191]
Human UCMSCs	Rats	Nerve injury-induced denervated muscle atrophy	Encapsulation in pH/ultrasound dual-responsive hydrogel	Muscle function recovery↑ Muscle atrophy↓ Fibrosis↓	[192]
Human SMSCs	Rats	Surgical-induced OA	——	Cartilage damage↓ Chondrocyte apoptosis↓	[193]
Human BMMSCs	Rats	Surgical-induced OA	Hypoxia preconditioning	Cartilage repair↑ Proteoglycan loss↓ OARSI score↓	[194]
Human SMSCs	Rats	Surgical-induced OA	Loaded with miR-140-5p	OA prevention↑ Chondrocyte proliferation↑ Cartilage tissue regeneration↑	[195]
Human UCMSCs	Rats	Osteochondral defect	Laden with DCM scaffold	Endogenous MSC chondrogenesis↑ Cartilage ECM deposition↑ Osteochondral repair↑ Subchondral bone regeneration↑	[196]

*MSC* mesenchymal stem cell, *EV* extracellular vesicle, *BMMSCs* bone marrow mesenchymal stem cells, *OVX* ovariectomy, *DPSCs* dental pulp stem cells, *mHA/PEG* mussel-inspired hyaluronic acid/polyethylene glycol, *IL6ST* interleukin 6 signal transducer, *UCMSCs* umbilical cord mesenchymal stem cells, *pH* potential of hydrogen, *SMSCs* synovial mesenchymal stem cells, *OA* osteoarthritis, *OARSI score* osteoarthritis research society international score, *DCM* decellularized cartilage extracellular matrix, *ECM* extracellular matrix

Mechanistically, BMMSC-EVs transport miR-335, which specifically targets and downregulates vesicle-associated membrane protein-associated protein B, thereby activating the Wnt/β-catenin signaling pathway and significantly enhancing osteogenic differentiation and bone defect repair [20]. In parallel, BMMSC-EVs deliver ubiquitin-specific protease (USP) 7, a deubiquitinating enzyme that stabilizes yes-associated protein (YAP) 1, leading to Wnt/β-catenin pathway activation. This mechanism not only promotes osteogenesis but also suppresses osteoclastogenesis, effectively restoring bone metabolic homeostasis [186]. Regarding angiogenesis, Nidogen1-enriched EVs derived from BMMSCs interact with myosin-10. This interaction disrupts focal adhesion formation and assembly, thereby reducing the adhesion strength between endothelial cells and the ECM. These changes collectively enhance endothelial cell migration and tube formation, thereby accelerating vascularization during bone regeneration [197]. Furthermore, recent studies demonstrate that SHED-EVs facilitate the mitochondrial translocation of dynamin-related protein 1 (Drp1). This translocation remodels mitochondrial dynamics and reverses the functional decline of BMMSCs induced by replicative senescence. Consequently, this process restores the innate pluripotency, immunomodulatory capacity, and therapeutic potential of these stem cells [198]. Additionally, MSC-EVs are enriched with Syntaxin 5 (STX5), an essential Golgi regulatory protein. STX5 effectively repairs damaged Golgi structures in senescent BMMSCs and restores vesicular secretion function. Ultimately, this significantly improves bone defect repair capacity while ameliorating osteoporotic phenotypes in aged mice [74]. Collectively, these findings highlight the promising potential of MSC-EVs in modulating endogenous stem cell senescence, facilitating angiogenesis-osteogenesis coupling, and achieving functional bone regeneration.

To enhance and sustain these therapeutic effects, various engineering strategies have been developed by researchers. These include hypoxic preconditioning to upregulate pro-angiogenic cargo, genetic modification such as BMP2 overexpression, and integration with biomaterials like β-tricalcium phosphate (β-TCP) or hydroxyapatite for sustained local release [199–202]. Notably, recent advances also encompass the engineering of MSC-ApoVs. Strategies such as surface modification with bone-targeting peptides or epigenetic reprogramming using histone deacetylases (HDAC) inhibitors, such as MI192, significantly enhance both osteogenic differentiation and bone-targeting capability [187, 203]. Beyond these approaches, the integration of engineered MSC-EVs with advanced hydrogels represents a particularly powerful strategy. A prominent example is the study by Wang *et al.*, in which DPSC-EVs derived from osteogenically induced cells were incorporated into a multifunctional, mussel-inspired adhesive hydrogel. When applied to a rat femoral condyle defect, this system demonstrated robust capabilities in promoting angiogenesis, accelerating mineralization, and facilitating substantial bone regeneration [188].

##### Regeneration of the skeletal muscle

Following skeletal muscle injury, tissue repair proceeds through interconnected inflammatory, regenerative, and remodeling phases that are coordinated by precise intercellular communication among immune cells, satellite cells, and stromal components [204]. During the inflammatory phase, neutrophils and macrophages are recruited to clear cellular debris and activate satellite cells, with anti-inflammatory macrophages subsequently facilitating myoblast differentiation and inflammatory resolution [204]. Recently, MSC-EVs have emerged as key mediators that exert beneficial therapeutic effects across all stages of the muscle repair process (Table 5).

During the initial inflammatory phase, MSC-EVs modulate the microenvironment by promoting macrophage polarization toward an anti-inflammatory phenotype and suppressing NF-κB signaling, thereby reducing fibrosis and improving muscle function *in vivo* [205, 206]. As regeneration proceeds, BMMSC-EVs enhance the muscle cross-sectional area, diminish fibrotic deposition, and increase capillary density [207]. In parallel, ADMSC-EVs deliver angiogenic proteins, such as platelet endothelial cell adhesion molecule (PECAM) and VEGF. When subjected to hypoxic conditions, these vesicles further enrich repair-associated miRNAs. Collectively, these molecular cargoes foster a pro-regenerative macrophage phenotype [205]. Notably, MSC-ApoVs enhance myoblast fusion and muscle regeneration by promoting creatine release. This vital release occurs through activated Pannexin 1 channels located on myoblast-derived ApoVs, revealing a novel metabolite-mediated mechanism of intercellular communication [189]. Furthermore, UCMSC-EVs promote peripheral nerve regeneration and muscle mass recovery by downregulating the pro-inflammatory cytokines IL-6 and IL-1β while upregulating the anti-inflammatory cytokine IL-10. Similarly, when administered post-contusion, BMMSC-EVs successfully reprogram macrophage polarization, attenuate fibrosis, and improve muscle contractile strength [190, 208].

The direct engineering of MSC-EVs or the specialized preconditioning of their parent MSCs can extensively refine cargo composition and targeting specificity. These modifications comprehensively amplify reparative target signaling within skeletal muscle. For example, researchers have developed engineered MSC-EVs that express a decoy receptor for the IL-6 signal transducer (IL6ST). These customized vesicles selectively inhibit IL-6 trans-signaling in muscle tissue while fully preserving classical IL-6 pathway activity [191]. Moreover, the hypoxic pretreatment of parent cells can modulate the molecular content of the resulting MSC-EVs. This environmental priming enhances regenerative outcomes and limits fibrotic responses [209]. Furthermore, the integration of MSC-EVs with tissue-engineered platforms enables sustained local release and improves skeletal muscle repair. In one illustrative case, an injectable hydrogel responsive to both ultrasound and potential of hydrogen (pH) triggers was designed to control the release of MSC-EVs. This responsive system effectively preserved muscle function in a rat denervation model by restoring muscle strength and mass while reducing inflammation and oxidative stress [192].

##### Osteoarthritis alleviation and cartilage protection

Osteoarthritis (OA) is a highly prevalent chronic joint disease, frequently originating from prior articular cartilage injury. The inherently limited regenerative capacity of cartilage tissue often leads to progressive degeneration, chronic pain, and functional impairment [210]. Pathophysiologically, OA is characterized by the disruption of chondrocyte homeostasis, resulting in an imbalance between ECM synthesis and degradation. Furthermore, this destructive process is accompanied by synovial inflammation, which synergistically accelerates terminal joint destruction [211]. As conventional surgical and cell-based therapies face considerable limitations, MSC-EVs have emerged as a promising cell-free alternative, offering reduced immunogenicity and simplified clinical implementation (Table 5) [210].

Building on this foundation, MSC-EVs have been shown to enhance chondrocyte viability, proliferation, and migration while promoting the synthesis of key ECM components such as aggrecan (ACAN) and collagen II. Concurrently, they suppress the expression of catabolic enzymes, including MMP-13 and a disintegrin and metalloproteinase with thrombospondin motifs-5 (ADAMTS-5) [212–215]. Notably, synovial MSC (SMSC)-EVs enriched with miR-140-5p promote matrix synthesis and attenuate OA progression in rat knee joints, partially through activation of the Hippo/YAP pathway [195]. Similarly, EV-derived miR-26a-5p from SMSCs, which targets phosphatase and tensin homolog, reduced apoptosis and inflammation in chondrocytes [193]. In a parallel mechanism, EV-derived miR-155-5p enhances their proliferation, migration, and ECM secretion [216].

Furthermore, preconditioning or engineering strategies applied to MSC-EVs heavily enhance their anti-catabolic and pro-chondrogenic effects in preclinical OA models. Prominent examples logically include hypoxic treatment or specific vesicular enrichment of miR-92a-3p as cargo [194, 217]. Notably, MSC-ApoVs have recently been identified as a novel and potent therapeutic entity. They orchestrate cartilage repair by delivering specific miRNAs, including miR-100-5p and let-7i-5p, which concurrently promote macrophage polarization toward the M2 phenotype and enhance endogenous chondrogenesis. When combined with decellularized cartilage ECM scaffolds, these vesicles demonstrate significant regenerative potential [196]. In parallel, the use of biomaterial-based delivery platforms, such as 3D printed ECM/gelatin-methacrylate scaffolds, has improved intra-articular retention and led to superior International Cartilage Repair and Joint Preservation Society (ICRS) scores compared to treatment with MSC-EVs alone [215]. Nevertheless, therapeutic efficacy may diminish in advanced or metabolically driven OA, and the standardization of EV isolation, dosing, and purity remains essential for clinical translation [218].

#### Regeneration of visceral organs

##### Cardiac repair

Cardiac repair progresses through a sequence of interconnected pathological stages, including acute inflammation, oxidative stress, programmed cardiomyocyte death, and fibrotic ECM remodeling. These factors collectively compromise pump function and limit endogenous regenerative capacity [219]. Furthermore, due to the limited proliferative potential of adult cardiomyocytes and the predominance of fibrotic scarring, conventional pharmacological or revascularization strategies cannot reverse established scarring or regenerate functional myocardium [219, 220]. In this context, MSC-EVs play a critical role in cardiac repair by reprogramming cardiac, vascular, and immune cells within the injured myocardium (Table 6).

**Table 6:** Functional properties of MSC-EVs in visceral organ regeneration

EV source	Species	Model	Engineering strategy	Outcomes	Ref.
Mouse BMMSCs	Mice	I/R injury	——	Infarct size↓ Cardiomyocyte apoptosis↓ Inflammation↓	[221]
Human UCMSCs	Mice	CVB3-induced viral myocarditis	——	Myocardial injury↓ Cardiac function↑ Cardiomyocyte apoptosis↓	[222]
Human UCMSCs	Mice	I/R injury	1. Loaded with miR-222 2. CTPs functionalization 3. Encapsulation into GelMA hydrogel	Cardiac function↑ Fibrosis↓ Cardiomyocyte apoptosis↓	[223]
Rat ADMSCs	Rats	Diabetic myocardial infarction	Enucleation and extrusion into mitochondria-containing EVs	Cardiac function↑ Fibrosis↓ M2 macrophage polarization↑	[224]
Human BMMSCs	Mice	HFD-induced diabetic liver injury	——	Hepatic steatosis↓ Liver insulin sensitivity↑ Liver macrophage infiltration↓	[225]
Human UCMSCs	Mice	CCl4-induced liver fibrosis	——	Liver fibrosis↓ Hepatic inflammation↓	[226]
Human UCMSCs	Mice	MCD diet-induced NAFLD	Diabetic microenvironment preconditioning	Lipid accumulation↓ Hepatocyte pyroptosis↓ Inflammation↓	[227]
Mouse BMMSCs	Mice	CCl4-induced ALF	1. Biomimetic mineralization with MnO2 2. Loaded with Dexamethasone	Oxidative stress↓ Hepatocyte ferroptosis↓ M1 macrophage polarization↓	[228]
Human UCMSCs	Mice	CLP-induced sepsis (with lung injury)	IL-1β preconditioning	Lung tissue injury↓ Macrophage infiltration↓	[229]
Human BMMSCs	Mice	Bleomycin-induced IPF	——	Pulmonary fibrosis↓ Collagen deposition↓ Myofibroblast activation↓	[230]
Human BMMSCs	Mice	LPS-induced acute lung injury	——	Lung tissue injury↓ Alveolar permeability↓ Inflammation↓	[231]
Human UCMSCs	Mice	Naturally aged pulmonary fibrosis	Anti-CD38 scFv functionalization	Pulmonary fibrosis↓ Senescent cell clearance↑ Metabolic function↑	[232]

*MSC* mesenchymal stem cell, *EV* extracellular vesicle, *BMMSCs* bone marrow mesenchymal stem cells, *I/R* ischaemia-reperfusion, *UCMSCs* umbilical cord mesenchymal stem cells, *CVB3* coxsackievirus B3, *CTPs* cardiac-ischemia-targeting peptides, *GelMA* gelatin methacryloyl, *ADMSCs* adipose-derived mesenchymal stem cells, *HFD* high-fat diet, *CCl4* carbon tetrachloride, *MCD diet* methionine- and choline-deficient diet, *NAFLD* nonalcoholic fatty liver disease, *ALF* acute liver failure, *CLP* cecal ligation and puncture, *IL-1β* interleukin-1β, *IPF* idiopathic pulmonary fibrosis, *LPS* lipopolysaccharide, *CD38* cluster of differentiation 38, *scFv* single-chain variable fragment

Accordingly, during the initial inflammatory phase, miR-181a derived from UCMSC-EVs suppresses c-FOS expression and attenuates inflammation induced by ischemia–reperfusion injury [233]. Concurrently, MSC-EVs promote macrophage polarization toward the anti-inflammatory M2 phenotypes *via* the S1P/sphingosine kinase 1 (SK1)/S1PR1 signaling axis [234]. Under conditions of oxidative stress, tissue inhibitor of metalloproteinases 2–enriched MSC-EVs enhance antioxidant capacity by increasing SOD and GSH levels, reducing MDA, and activating the AKT/secreted frizzled-related protein 2 pathway. Meanwhile, MSC-EVs support myocardial ATP production and inhibit c-JNK phosphorylation by promoting the phosphorylation of AKT and glycogen synthase kinase-3beta [235, 236]. In the regulation of cell death, miR-25-3p carried by MSC-EVs suppresses the Fas ligand/phosphatase and tensin homolog pathway to reduce cardiomyocyte apoptosis, while miR-182-5p downregulates gasdermin D to limit pyroptosis [221, 237]. Furthermore, MSC-EVs activate AMPK and inhibit mechanistic target of rapamycin (mTOR) to enhance autophagy in viral myocarditis models [222]. During the remodeling phase, MSC-EV-derived miR-125a-3p targets transforming growth factor beta receptor 1 to inhibit fibroblast activation and attenuate cardiac fibrosis [238]. Beyond suppressing adverse remodeling, MSC-EVs actively promote reparative processes such as angiogenesis. Notably, Liu *et al.* demonstrated that ApoVs from transplanted MSCs are engulfed by endothelial cells, where they activate TFEB-mediated lysosomal biogenesis and autophagy. This enhanced autophagic flux subsequently stimulates angiogenesis *via* the AKT/nitric oxide synthase (NOS) 3 pathway, significantly contributing to functional recovery after myocardial infarction [239].

Based on these intrinsic therapeutic properties, engineered MSC-EVs and their biomaterial composites demonstrate enhanced efficacy in cardiac repair. For instance, researchers have developed miR-222-engineered EVs functionalized with cardiac-ischemia-targeting peptides and encapsulated within gelatin methacryloyl (GelMA) hydrogels [223]. This engineered system improves myocardial repair by enhancing cellular uptake and activating mechanotransduction pathways. Similarly, mitochondria-enriched MSC-EVs facilitate macrophage polarization toward the M2 phenotype, restore mitochondrial function, and ameliorate cardiac dysfunction in diabetic myocardial infarction models [224]. Moreover, a biomimetic and NOS-responsive nanomotor has been developed from hybrid MSC and macrophage membranes [240]. This system is loaded with a mitochondrial ROS scavenger to enable deep tissue targeting. It alleviates oxidative DNA damage, restarts cardiomyocyte proliferation, and significantly improves cardiac function after ischemia–reperfusion injury.

##### Liver regeneration

Following acute or chronic hepatic injury, liver repair typically progresses through three distinct phases. The first is an initial inflammatory phase dominated by Kupffer cells, dendritic cells, natural killer cells, and natural killer T cells within the sinusoidal niche. This is followed by a subsequent resolution phase and, finally, a proliferative remodeling stage that restores parenchymal structure and function [241]. However, excessive oxidative stress and damage-associated molecular patterns amplify JNK activation and NLRP3 inflammasome signaling, which perpetuate hepatocyte death and impair endogenous regenerative capacity [242]. In this context, MSC-EVs represent a novel cell-free platform characterized by inherent liver tropism and low immunogenicity, making them a promising therapeutic strategy for promoting liver regeneration after injury (Table 6).

Aligned with the hepatic repair process, MSC-EVs exert multifaceted effects during the inflammatory phase. They achieve this by promoting the polarization of hepatic macrophages toward anti-inflammatory phenotypes, suppressing the LPS/TLR4 signaling axis, and reducing levels of pro-inflammatory cytokines including TNF-α, IL-1β, and IL-6 [243, 244]. Notably, MSC-ApoVs are efferocytosed by liver macrophages *via* surface-exposed calreticulin, thereby restoring macrophage homeostasis and attenuating chronic inflammation [225]. During the resolution phase, MSC-EVs mitigate hepatocyte apoptosis and enhance autophagic flux, partly through the delivery of glutathione peroxidase (GPX) 1, which facilitates oxidative stress control and promotes cytoprotection in acute liver injury [245, 246]. Simultaneously, they modulate the crosstalk between hepatic stellate cells and macrophages by delivering EV-derived miR-148a. This specific miRNA targets the Krueppel-like factor (KLF) 6/signal transducer and activator of transcription 3 (STAT3) pathway in macrophages, thereby alleviating fibrotic barriers to tissue regeneration [226]. Finally, during the proliferative phase, MSC-EVs promote parenchymal repopulation by upregulating proliferative markers such as PCNA and cyclin D1, and by transiently inducing a hepatocyte-to-progenitor-like (oval cell) transition [247, 248]. Furthermore, MSC-ApoVs assemble with the hepatocyte Golgi apparatus to form a chimeric organelle complex that promotes microtubule acetylation and cytokinesis, thereby significantly enhancing liver regeneration [91].

To further enhance liver repair, researchers have recently developed multiple engineered and composite delivery strategies. For instance, preconditioning MSCs in a diabetic environment yields EVs that suppress hepatocyte pyroptosis through the action of Peroxiredoxin-1 (PRDX-1) [227]. In parallel, genetically modified MSC-EVs engineered to overexpress USP10 ameliorate liver fibrosis by reprogramming macrophages *via* the KLF4/MMP12 axis [249]. Additionally, a biomimetic mineralized system comprising MSC-EVs surface-modified with MnO_2_ and loaded with dexamethasone was developed to alleviate ferroptosis and inflammation *via* ROS scavenging and stabilization of the solute carrier family 7 member 11/GPX4 pathway [228]. More recently, a sustained-release hydrogel encapsulating aminoethyl anisamide–functionalized MSC-EVs has been shown to mitigate hepatic fibrosis by reducing oxidative stress and collagen deposition [250]. Together, these multifaceted engineering and delivery strategies underscore the potential of integrated platforms for precise and effective liver regeneration.

##### Pulmonary repair

Pulmonary repair following injury progresses through distinct phases. It begins with the initial disruption of the alveolar-capillary barrier, which is characterized by protein-rich edema and inflammatory cell infiltration. This is followed by a proliferative stage and, if dysregulated, a gradual progression to fibrotic remodeling [251]. Persistent injury is driven by epithelial apoptosis, dysregulated autophagy, endothelial hyperpermeability, macrophage activation, and neutrophil extracellular trap formation [252]. Consequently, effective interventions must restore epithelial and endothelial integrity while rebalancing innate immune responses. In this context, MSC-EVs exhibit the ability to modulate inflammation, enhance alveolar epithelial regeneration, repair microvascular permeability, and attenuate fibrogenesis, while being more stable and less immunogenic than their parent cells (Table 6) [221–232].

MSC-EVs actively engage in repair mechanisms across these pathological stages. During the initial injury phase, they attenuate inflammatory cytokine cascades and reprogram innate immune responses. For instance, MSC-EVs deliver specific miRNAs, including miR-146a and miR-27a-3p, which promote macrophage polarization toward an anti-inflammatory M2 phenotype [229, 231]. Similarly, MSC-ApoVs suppress inflammation by engaging the PDL1-PD1 axis to shift macrophage metabolism from glycolysis to oxidative phosphorylation, thereby inducing an anti-inflammatory phenotype [253]. Concurrently, MSC-EVs reduce early neutrophil infiltration into lung tissue and decrease levels of macrophage inflammatory protein-2 (MIP-2) in bronchoalveolar lavage fluid. They achieve this through the delivery of cargo such as keratinocyte growth factor mRNA [254]. At the pulmonary epithelial interface, MSC-EVs exert protective effects by upregulating G Protein-coupled receptor class C group 5 member A to inhibit apoptosis and restore barrier integrity. They also induce autophagy and transfer functional mitochondrial DNA to preserve cellular function [255–257]. In the pulmonary microvascular endothelium, MSC-EVs enhance barrier function by delivering angiopoietin-1 mRNA and hepatocyte growth factor, which upregulate junctional proteins. Simultaneously, they suppress hyperpermeability by inhibiting the transient receptor potential vanilloid 4/Ca^2+^ pathway [258–260]. During the remodeling phase, MSC-EVs attenuate fibrosis by modulating fibroblast activation through the delivery of miR-186 and miR-29b-3p. These miRNAs target SRY-related HMG box transcription factor 4, dickkopf-1, and frizzled 6, respectively [230, 261].

To further enhance the therapeutic efficacy of MSC-EVs, engineered variants have been developed. For example, MSC-EVs modified with the CD38 antigen receptor specifically target senescent alveolar epithelial cells, restoring nicotinamide adenine dinucleotide (NAD^+^) levels and reversing epithelial-mesenchymal-transition in pulmonary fibrosis models [232]. In parallel, integrating EVs with mucoadhesive biomaterials, such as chitosan or hyaluronic acid that electrostatically anchor to airway mucus, prolongs retention time and enhances resistance to mucociliary clearance [262]. Additionally, smart responsive materials enable the on-demand EV release in inflammatory microenvironments [262]. Clinically, nebulized UCMSC-EVs have demonstrated favorable safety profiles and significantly improved pulmonary function parameters, including forced vital capacity and quality of life scores, underscoring the translational potential of MSC-EVs for precision pulmonary repair [263].

Collectively, the therapeutic applications discussed in Section 2.2 demonstrate that MSC-EVs orchestrate tissue repair across orofacial, barrier, musculoskeletal, and visceral systems through a conserved set of core mechanisms. These mechanisms include immunomodulation, angiogenesis, anti-fibrosis, the activation of endogenous stem cells, the restoration of barrier integrity, and the regulation of cell death pathways. These shared biological properties, coupled with their low immunogenicity and inherent amenability to engineering modifications, establish MSC-EVs as a versatile and adaptable platform for regenerative medicine. However, the clinical translation of these promising preclinical findings is contingent upon overcoming common hurdles. The most notable challenges include the inherent heterogeneity of natural MSC-EVs, their limited targeting specificity, and the urgent need for scalable and standardized manufacturing processes with rigorous QC. These challenges underscore the necessity for the advanced engineering strategies and clinical translation frameworks discussed in the following sections.

### Engineering modification and clinical translation of MSC-EV therapy

#### Clinical translation bottlenecks of natural MSC-EVs

Numerous studies have underscored the substantial promise of natural MSC-EVs in tissue repair and disease treatment. However, several challenges need to be addressed before they can be successfully translated into clinical applications. A primary concern is heterogeneity. MSC-EVs constitute a highly heterogeneous population encompassing EXOs, MVs, and ApoVs, which differ in size, biogenesis, and biological functions. Moreover, variations in tissue sources, inter-donor differences, and culture conditions considerably affect the proteomic and miRNA profiles of MSC-EVs [264–269]. These factors contribute to batch-to-batch variability in therapeutic efficacy, hampering the consistency and reproducibility required for clinical applications. Another key challenge is the limited inherent homing capacity of natural MSC-EVs. Following systemic administration, the majority are rapidly cleared by the mononuclear phagocyte system in the liver and spleen, resulting in < 1% reaching the target tissue [10]. Furthermore, natural MSC-EVs often exhibit suboptimal cargo composition. Specifically, they lack the optimally coordinated molecular combinations necessary for synergistic effects, and they may concurrently carry both therapeutic and undesirable components. These limitations collectively lead to restricted and often unpredictable biological outcomes [270]. In response to these limitations, engineered MSC-EVs have emerged as a promising next-generation strategy to overcome the shortcomings of their natural counterparts [271, 272]. Through purposeful molecular and functional modifications, engineered MSC-EVs can overcome challenges related to heterogeneity, limited targeting, and suboptimal therapeutic cargo, thereby enabling the rational design of EVs with enhanced and more predictable therapeutic performance [273].

#### Engineering strategies: precise manipulation from internal loading to surface modification

**Figure 3:**
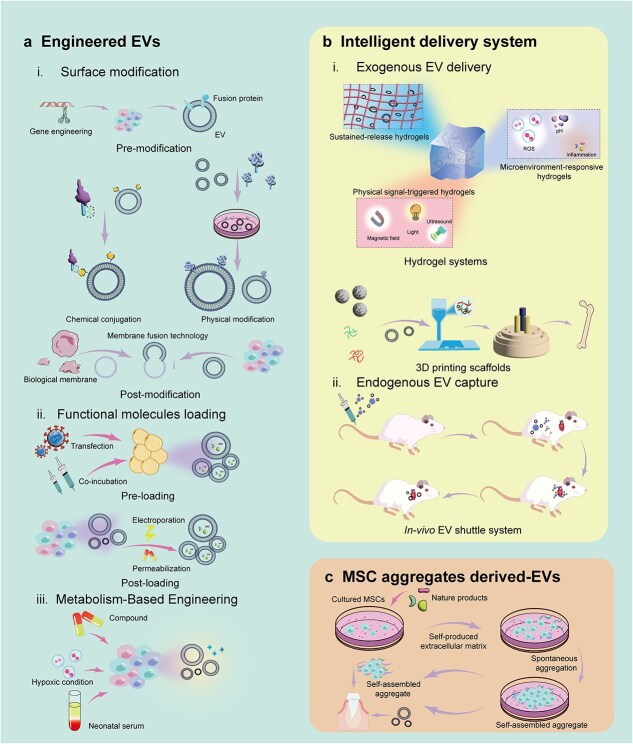
Engineering strategies for MSC-EV-based regenerative medicine. (**a**) Commonly employed engineering modification of EVs through surface modification (i), functional molecules loading (ii), and metabolic reprogramming (iii) in regenerative applications. (**b**) Intelligent delivery systems for EVs utilizing biomaterials for exogenous EVs (i) and engineered particles for endogenous EV capture (ii). (**c**) Engineered MSC aggregate-derived EVs strategy based on organ morphogenesis principles. *EV* extracellular vesicle, *ROS* reactive oxygen species, *pH* potential of hydrogen, *3D* three-dimensional

Engineered EVs are natural EVs that have been artificially modified through biological, physical, or chemical strategies. These modifications enhance the ability of EVs to specifically recognize and target recipient cells or tissues, thereby enabling the precise delivery of encapsulated therapeutic cargo. This strategy holds substantial promise for a wide range of biomedical applications [274]. Commonly employed engineering approaches include surface modification, therapeutic cargo loading, and the metabolic reprogramming of EVs (Figure 3a).

##### Surface modification of engineered MSC-EVs

Surface modification involves the design and incorporation of functional ligands onto the EV membrane, enabling specific recognition and binding to receptors on target cells [275–277]. Pre-secretion modification entails genetically or functionally engineering donor cells so that the resulting EVs inherit the desired modifications through natural biogenesis or artificial preparation. The principal approach is genetic engineering, wherein a target protein is expressed as a fusion partner with an endogenous membrane protein, leading to its display on the EV surface. For instance, Wang *et al.* fused an ischemic myocardium-targeting peptide with the exosomal membrane protein lysosomal associated membrane protein 2B (LAMP2B). The resulting engineered MSC-EVs exhibited significantly enhanced accumulation in ischemic myocardial tissue and promoted cardiac repair and regeneration [276].

In contrast, post-secretion modification employs simpler mechanisms and offers broader applicability. This strategy involves directly anchoring exogenous functional molecules onto isolated EVs *via* physical adsorption or chemical conjugation. Chemical conjugation relies on reactions between functional modules and chemical groups present on EV membrane proteins. Click chemistry, particularly the azide-alkyne cycloaddition using dibenzocyclooctyne (DBCO), is widely adopted due to its rapid kinetics and high efficiency [278, 279]. In this approach, azide or alkyne groups are introduced onto isolated EVs, which allows for covalent binding to complementary functional molecules, thereby achieving surface functionalization [280]. For example, Tian *et al.* conjugated azide-modified cyclo (Arg-Gly-Asp-D-Tyr-Lys) peptide [c(RGDyK)] peptides to DBCO-modified MSC-EVs, yielding vesicles that targeted cerebrovascular endothelial cells with high affinity and showed therapeutic efficacy in models of ischemic brain injury and stroke [281].

The most common physical modification method is hydrophobic insertion, in which ligands are covalently conjugated to hydrophobic anchors to create amphiphilic molecules. When incubated with pre-isolated EVs, the hydrophobic moieties of these conjugates spontaneously insert into the lipid bilayer of EV membranes, displaying the linked functional ligands on the vesicle surface. In a representative application, Gu *et al.* conjugated the cardiomyocyte specific peptide with 1,2-distearoyl-sn-glycero-3-phosphoethanolamine-N-[hydroxysuccinimidyl (polyethylene glycol)-2000] and inserted this construct into BMMSC-EV membranes. The resulting engineered EVs effectively delivered miR-302 to cardiomyocytes with enhanced cardiac targeting, providing a novel therapeutic strategy for alleviating myocardial ischemia–reperfusion injury [282].

Although surface modification techniques enhance the targeting specificity of EVs, they may interfere with natural cargo sorting, alter membrane properties, and potentially induce immunogenic responses or nonspecific adsorption [283–285]. To overcome these limitations, research has shifted toward biomimetic internalization strategies. In this context, biohybrid vesicles, fabricated through membrane fusion technology, have emerged as a promising alternative. These vesicles integrate biological membranes with homologous or heterologous components to achieve functional surface properties [286]. For instance, Li *et al.* fabricated biohybrid nanovesicles by fusing platelet membranes with MSC-EVs *via* co-extrusion. The resulting vesicles retained the innate targeting ability of platelets toward injured cardiac tissue while inheriting the MSC-derived functions of vascular repair and angiogenesis promotion. This membrane fusion strategy is not limited to natural biological membranes but can also be extended to synthetic systems [287]. In another study, Chen *et al.* fused MSC membranes with cyclosporine A-loaded liposomes through co-extrusion. The resulting hybrid vesicles preserved the complete proteomic profile of MSC membranes, including key functional proteins such as C-X-C chemokine receptor type 4 and very late antigen-4, conferring inflammatory targeting and immune evasion capabilities. Moreover, these vesicles maintained the high drug-loading capacity and sustained-release characteristics of liposomes [288]. In summary, biohybrid vesicles represent a successful integration of bionic principles and engineering design, creating a versatile nanoplatform that combines the complex biological functionality of natural membranes with the controllable drug delivery and high payload capacity of synthetic carriers.

##### Functional molecule loading of engineered MSC-EVs

Functional molecule loading refers to the process of encapsulating specific small-molecule drugs, nucleic acids, and proteins within EVs [289]. The pre-secretion loading of functional molecules (pre-loading) primarily employs two approaches: co-incubation and transfection [290]. Co-incubation relies on passive uptake, in which donor cells are incubated with functional molecules, leading to their spontaneous encapsulation into EVs during biogenesis. Transfection, in contrast, utilizes genetic engineering to directly modify donor cells, enabling the resulting EVs to carry and deliver the desired molecular cargo. In our previous work, we combined the adenoviral transduction of the osteogenic factor RING finger protein146 into BMMSC-ApoVs with surface modification using a bone-targeting peptide (Asp-Ser-Ser)_6_ ((DSS)_6_). This engineered EV system significantly promoted bone regeneration in osteoporosis models [187]. For EVs that have been isolated and purified from donor cells, therapeutic agents can either be associated with the EV membrane or encapsulated within the lumen using passive or active loading strategies. These techniques are collectively referred to as post-loading [291]. Passive loading typically relies on simple co-incubation, whereas active loading employs physical or chemical techniques to transiently enhance membrane permeability, thereby facilitating drug entry. Commonly used active methods include electroporation, sonication, extrusion, repeated freeze–thaw cycles, chemical agents mediated permeabilization, and transfection-based approaches [292–294].

Pre-loading and post-loading strategies differ significantly in their biological properties. Pre-loading maintains the native membrane architecture of EVs, preserving their superior biocompatibility and inherent targeting capabilities, which are particularly crucial for regenerative applications requiring complex cell–cell communication. In comparison, post-loading offers greater engineering flexibility, but the requisite physical or chemical treatments can compromise membrane integrity. This may result in cargo leakage and the denaturation of surface-targeting proteins, ultimately diminishing the vesicle’s biological functionality. Consequently, pre-loading is the preferred choice for investigating intrinsic EV functions or conducting complex signaling studies in basic research, whereas post-loading provides superior engineering flexibility for targeted drug delivery applications.

##### Metabolism-based engineering strategy for EVs

Metabolic modification involves adjusting the culture conditions of parent cells to regulate their activity and metabolic state, thereby indirectly modulating the production and composition of EVs. For instance, SHED-derived EVs produced under hypoxic conditions demonstrate enhanced potential in promoting angiogenesis and osteogenesis, showing a 1.6-fold increase in OCN expression and 1.4-fold increase in calcium deposition *in vitro*, as well as a 95% increase in bone volume *in vivo* [295]. Similarly, EVs harvested from metformin-treated MSCs alleviate mitochondrial dysfunction and impaired autophagy in senescent cells through the remodeling of energy metabolism, consequently improving wound healing in aged mouse models [296]. While such strategies rely on primarily exogenous metabolic interventions to modify cellular metabolism, naturally occurring metabolic reprogramming signals within complex physiological environments can also guide cells to secrete therapeutically potent EVs [297]. For example, preconditioning MSCs with neonatal serum-derived EVs can precisely modulate the metabolic pathways of parent cells, reverting the cells to a more juvenile state with enhanced regenerative capacity [298]. The EVs subsequently released by these rejuvenated MSCs exhibit significantly improved pro-angiogenic activity and accelerate cutaneous wound healing. Looking forward, future advances will focus on achieving more precise and intelligent metabolic engineering of EVs, likely involving the use of metabolomic profiling to identify specific metabolite signatures responsible for functional EV properties, combined with machine learning approaches for the targeted design and predictive functional optimization of EV-based therapeutics.

Beyond these preconditioning approaches, there is growing interest in directly targeting specific metabolic pathways in MSCs to enable more precise engineering of EV function. Preliminary investigations offer initial clues, though direct evidence remains limited. One study found that inhibiting oxidative phosphorylation in MSCs diminished the pro-angiogenic activity of their EVs, while the inhibition of glycolysis had no significant effect [299]. Another report showed that exposing MSCs to advanced glycation end products modulated the miRNA cargo of their EVs, with differential miRNA expression linked to glycolysis and tricarboxylic acid cycle pathways, and these EVs subsequently induced metabolic changes in recipient endothelial cells [300]. These findings suggest a potential link between MSC metabolic states and EV functional properties, highlighting the need for further investigation.

#### Intelligent delivery systems: Achieving accurate release in time and space

The limited tissue retention and rapid systemic clearance of EVs have prompted the development of advanced delivery systems using bioactive materials (Figure 3b). These systems are designed to enhance spatiotemporal control over EV delivery, thereby improving their bioavailability and therapeutic efficacy in target tissues [301]. By achieving more precise targeting, EVs can exert their therapeutic effects more efficiently at the desired site, particularly in applications such as anti-inflammatory and immunomodulatory therapies. Optimizing these systems not only improves the delivery efficiency but also minimizes potential side effects, thereby enhancing overall therapeutic outcomes.

##### Hydrogel systems

Hydrogels have emerged as ideal carriers for EV delivery owing to their excellent biocompatibility, biodegradability, and tunable structural properties, which facilitate the controlled release of EVs at target sites [302–304]. Based on their release mechanisms, hydrogels can be classified into three categories: sustained-release, microenvironment-responsive, and physical signal-triggered systems [305–307]. Sustained-release hydrogels function by modulating the crosslinking density and pore architecture of the hydrogel matrix, thus achieving gradual and prolonged EV release as the material undergoes degradation [308]. This type is particularly suitable for long-term regenerative applications, such as chronic wound healing and post-infarction myocardial repair. Microenvironment-responsive hydrogels are engineered to react to pathological signals, such as acidic pH, elevated ROS, or inflammatory markers [309, 310]. For example, Li *et al.* developed an intelligent ROS-responsive hydrogel based on a RhB-AC monomer, which exhibits high selectivity for hypochlorous acid (HClO/ClO^−^), which is a key oxidizing agent and inflammatory mediator in pulpitis. The oxidation of the RhB-AC component triggers controlled hydrogel degradation, resulting in the sustained release of dental follicle-derived MSC-EVs. Compared to free EV administration in pulpitis models, this delivery system reduces DNA oxidative damage (8-OHdG^+^CD90^+^ cell ratio) by 40%, increases reparative dentin volume by 50%, and lowers inflammatory scores by 33% [311]. Physical signal-triggered hydrogels utilize external stimuli such as light, ultrasound, or magnetic fields to achieve spatiotemporally controlled EV release [312]. A representative example is the GelMA-based hydrogel developed by Liu *et al.*, which incorporates UCMSC-EVs and Fe₃O₄@BaTiO₃ core–shell nanoparticles. The key innovation of this magnetic hydrogel lies in its Fe₃O₄ core, which converts externally applied magnetic energy into mechanical vibrations. These vibrations are efficiently transduced into localized microcurrents *via* the piezoelectric BaTiO₃ shell, enabling remote and non-invasive magnetoelectric stimulation. This approach acts synergistically with the immunomodulatory functions of the loaded EVs, effectively suppressing early inflammatory responses, promoting neurogenesis and axonal regeneration in spinal cord injury models, and ultimately facilitating functional neural recovery [313].

##### 3D printed scaffolds

3D printed scaffolds can synergistically remodel the regenerative microenvironment with EVs through structural and material design, thereby facilitating efficient, safe, and durable tissue regeneration. In a study by Zheng *et al.*, EVs isolated from growth differentiation factor-5 (GDF-5)-preconditioned SMSCs were incorporated into a photocrosslinkable 3D-bioprinted glycyrrhizic acid (GA)/hyaluronic acid (HA) scaffold. This composite scaffold not only leverages the anti-inflammatory properties of GA but also protects the EVs from enzymatic degradation within the joint cavity, thereby enhancing the repair of cartilage defects [314]. Furthermore, 3D printed scaffolds can be customized to conform to the spatial architecture of the defect site. For example, Jiang *et al.* fabricated a bionic scaffold that mimics natural microchannel structures and cortical bone networks. They then immobilized engineered ADMSC-EVs onto the scaffold, thus establishing a local microenvironment conducive to vascularization and osteogenesis within the defect area [315].

Beyond directly loading exogenous EVs onto 3D-printed scaffolds, emerging strategies are exploring ways to actively recruit endogenous EVs to the scaffold interface. For instance, Liu *et al.* developed an innovative ‘*in vivo* EV shuttle system’ based on CD63 antibody-conjugated magnetic nanoparticles, which captures circulating EVs from the host serum and releases them under acidic conditions [316]. When integrated into a scaffold system, such a strategy could enable the scaffold not only to serve as a structural support but also to actively recruit and concentrate endogenous EVs carrying reparative signals. Combining EV-capturing nanoparticles with 3D-printed scaffolds could create a dual-mode platform capable of both the active recruitment and localized release of EVs, laying the foundation for a new generation of ‘intelligent regenerative scaffolds’ that detect and respond to the disease microenvironment.

#### Engineered MSC aggregate-derived EVs based on organ morphogenesis principles

Although significant progress has been made in developing engineered vesicle strategies to enhance function, most methods still rely on exogenous interventions. These methods are limited by factors such as high procedural complexity, potential risks associated with the introduction of external components, and a lack of multidimensional functional regulation. Tissue and organ regeneration are complex processes that involve the spatiotemporal coordination of multiple developmental signals, and their regulatory mechanisms often surpass the capabilities of any single engineering strategy. During embryonic development, mesenchymal condensation is a critical event in organ morphogenesis [317, 318]. In this process, MSCs maintain stemness, exhibit multi-directional differentiation potential, and regulate spatiotemporal gene expression through cell self-assembly, mechanical contraction, and ECM remodeling [318–321].

Inspired by the self-organizing and regenerative properties of MSCs, our team has introduced an innovative strategy for constructing stem cell aggregate-derived vesicles (CA-EVs) [322] (Figure 3c). Unlike traditional exogenous intervention methods, this approach induces adult MSCs to form aggregates, simulating mesenchymal condensation, activating intrinsic developmental and regenerative programs, and stimulating the MSCs to naturally produce and release vesicles enriched with physiological developmental signaling molecules. These CA-EVs possess excellent biocompatibility and signal integration capabilities, making them ideal mediators for developmental regeneration and providing a novel research approach for the functional reconstruction of complex tissues and organs.

Regarding the engineered construction of MSC aggregates, conventional methods often use ultra-low attachment plates or AggreWell microplates to allow high-density cells to spontaneously aggregate into spheres through surface tension and gravity [323–325]. Additionally, bioactive materials like hydrogels can serve as scaffolds to promote close cell contact and aggregate formation by physically confining cells within a narrow space [326–328]. However, these methods rely heavily on external regulation. Moreover, due to spatial constraints and short culture durations, ECM deposition is limited, which creates a significant gap compared to the ECM abundance found in physiological mesenchymal condensation [329, 330]. To overcome this limitation, our team has developed a self-assembled cell sheet aggregate technology, which facilitates the formation of cell sheets rich in natural ECM through self-assembly [331–333]. After gentle detachment and 3D shaping, MSC aggregates that mimic physiological mesenchymal condensation are constructed. This method maximizes the retention of autocrine ECM, effectively promoting cell survival, stemness, and lineage differentiation. Furthermore, cell sheets derived from various sources, such as PDLSCs and BMMSCs, can be stacked to create a biomimetic ‘regenerative cementum-periodontal ligament-alveolar bone interface,’ offering a novel solution for complex tissue repair [334, 335].

Notably, compared to monolayer culture systems, MSC aggregate culture significantly improves both the production efficiency and functional activity of CA-EVs. This is primarily reflected in the enhanced paracrine function of the cells [120, 167]. For example, in skin regeneration studies, CA-EVs derived from UCMSC aggregates exhibit a 2.5-fold increase in protein content and a 3.2-fold increase in particle number compared to those from monolayer cultures [167]. In subsequent tooth regeneration studies, specific microenvironment have been shown to play a crucial role in amplifying vesicle release. For instance, in the odontogenic microenvironment provided by DTM, SHED aggregates release ~3.2 times more vesicles [120]. These findings confirm that aggregate culture effectively promotes MSC vesicle release, and the synergistic effects of specific microenvironments can further enhance this process, thereby providing strong experimental evidence for optimizing vesicle yield and function.

At the molecular level, aggregate-derived EVs exhibit significant functional enhancement by having optimized miRNA and protein cargo compositions. In dental pulp regeneration, CA-EVs are enriched in miR-26a, which activates the TGF-β/Smad2/3 signaling pathway to promote angiogenesis [97]. In skin wound repair, CA-EVs are rich in pro-angiogenic proteins such as nicastrin and presenilin-1, which activate the Notch signaling pathway and specifically induce the generation of regeneration-associated vascular subtypes. Functional verification shows that CA-EVs possess strong capabilities in promoting endothelial differentiation, vascular network formation, and tissue regeneration both *in vitro* and *in vivo*. They can also reverse the inhibitory effects of pathological conditions, such as high glucose, on angiogenesis, thus providing key experimental evidence for the development of efficient cell-free regenerative therapies [167].

Moreover, CA-EVs play a crucial role in mobilizing and restoring the function of endogenous stem cells, supporting efficient tissue regeneration [74, 127]. Specifically, CA-EVs can mobilize quiescent or *in situ* endogenous stem cells and restore the activity of aged or functionally impaired stem cells. In periodontal tissue regeneration, CA-EVs successfully activate host endogenous Gli1^+^ stem cells to promote tissue regeneration [127]. In bone tissue regeneration, CA-EVs carry key proteins such as STX5, which repair the Golgi apparatus in aged BMMSCs, restoring their self-renewal and osteogenic differentiation capabilities. This ultimately promotes bone defect healing and improves osteoporosis [74]. In conclusion, CA-EVs constructed using these engineered strategies not only significantly increase vesicle yield but also comprehensively optimize their quality, function, and therapeutic potential by mimicking the mesenchymal condensation microenvironment. This strategy offers distinct advantages over traditional MSC-EVs in regenerative medicine, providing a novel approach to the functional reconstruction of complex tissues and organs.

### Advancing to clinical practice

#### Scalable production processes

MSC-EVs demonstrate considerable therapeutic promise across various pathological conditions, including tissue and organ injuries, inflammatory disorders, and age-related degenerative diseases. This broad applicability highlights their potential in regenerative medicine. However, translating these results into reliable clinical applications presents a significant challenge, particularly in achieving stable and scalable production that meets the stringent QC requirements for therapeutic use [336, 337].

##### Technical challenges and process optimization for large-scale production

**Figure 4:**
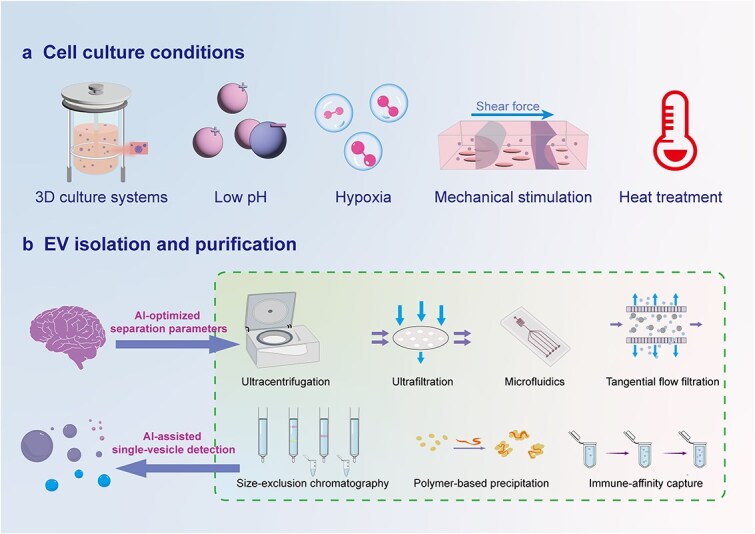
Achieving scalable production of MSC-EVs through advanced upstream and downstream strategies. This schematic illustrates the key manufacturing stages for MSC-EVs and highlights the critical path toward achieving scalable and consistent EV production for clinical applications. (**a**) Optimization of culture conditions to enhance MSC-EV yield. (**b**) Techniques and AI integrated strategies for EV purification and analysis. *3D* three-dimensional, *pH* potential of hydrogen, *AI* artificial intelligence

The manufacturing process for MSC-EVs comprises multiple stages, including cell culture, EV secretion, isolation, purification, formulation, filling, final product preparation, storage, and distribution [338]. Optimizing EV yield begins with enhancing the cell culture conditions (Figure 4a). In industrial-scale production, 3D culture systems provide larger surface areas and better simulate *in vivo* environments, which have been reported to increase EV yields by 19.4-fold compared to conventional two-dimensional (2D) cultures [339]. The composition of the culture medium and environmental parameters also influence EV secretion. For instance, serum-free media minimize contamination risks and can upregulate EV secretion [340]. Specific conditions like low pH, hypoxia, and mechanical stimulation have also been reported to enhance EV production [341–344]. However, the efficacy of these strategies remains variable and unreliable due to the inherent heterogeneity among cellular sources and the complex, nonlinear regulation of EV secretion by culture conditions [345–347].

The isolation and purification of EVs are critical steps for enhancing both sample purity and concentration (Figure 4b). Commonly employed techniques include ultracentrifugation, tangential flow filtration (TFF), UF, SEC, and polymer-based precipitation [339]. These methods separate EVs based on physical characteristics such as size, density, and solubility, offering broad applicability while helping to retain the native biological features of the EVs. However, a key trade-off persists: methods with high recovery rates often yield lower purity, whereas high-purity methods frequently suffer from limited recovery. Consequently, hybrid strategies that combine multiple physical separation principles have become mainstream. For instance, Liu *et al.* developed a fully automated platform named ‘FACTORY’ for EV isolation, which integrates continuous-flow centrifugation with TFF. This system enables the automated, standardized, and high-quality isolation of UCMSC-EVs from 10-liter samples, achieving a 3-fold higher yield than conventional methods while significantly reducing inter-batch variability [348]. Nevertheless, these methods primarily enrich EVs that share similar physical properties without ensuring consistency in their biological function [349]. For applications requiring high specificity, immune-affinity capture provides an alternative by selectively isolating EVs through antibody–antigen interactions [350]. Another promising technology is microfluidics, which enables precise fluid manipulation through microfabricated structures [351, 352]. Microfluidic systems can integrate multiple separation mechanisms (*e.g.* physical and affinity-based methods) and combine sample processing, isolation, purification, and downstream detection into a single platform. In summary, no single isolation method is universally applicable, and scalable EV production strategies must be rationally designed for specific clinical applications.

##### AI-optimized manufacture for intelligent scaled production of EVs

In the face of the complex and nonlinear challenges inherent in production processes, AI offers a novel technological pathway to address the issues of standardization and control in the large-scale production of MSC-EVs [353]. Through data-driven approaches, AI is transforming the process optimization, which was previously reliant on experience, into a predictable and controllable intelligent production flow [354, 355]. In the critical phase of large-scale production, particularly regarding the isolation and QC of MSC-EVs, AI can substantially enhance the standardization and efficiency of the preparation process through data-driven approaches. For example, AI can dynamically optimize the operating parameters of critical separation equipment, such as ultracentrifugation and TFF. This optimization improves EV purity and yield while maximizing vesicle integrity and bioactivity to meet the GMP standards required for the large-scale production of therapeutic EVs [356]. In the characterization phase, the integration of deep learning architectures with rapid detection technologies, such as surface-enhanced Raman scattering, enables the high-throughput, non-destructive analysis of individual EV surface markers [357]. This has the potential to replace traditional, time-consuming, and less-reproducible manual methods, thereby serving as a powerful tool for online quality monitoring. Additionally, the development of AI-based probabilistic models and intelligent affinity separation strategies allows for the efficient and accurate capture of target EVs from complex samples, providing the technological foundation for the production of high-purity EV formulations [358, 359].

In terms of functional enhancement, AI provides new approaches for the precise engineering of MSC-EVs, which is crucial for producing standardized EV products with specific therapeutic functions. By simulating the interactions between MSC-EVs and specific tissue cells, AI can predict and optimize their natural tissue-homing ability, thereby guiding the design of more efficient targeting strategies [360]. Machine learning algorithms can also be applied to the large-scale screening of specific targeting peptides or the optimization of therapeutic molecule loading schemes, thereby selectively enhancing MSC-EVs therapeutic efficacy in specific tissue regeneration contexts. In the areas of mechanism analysis and therapeutic efficacy evaluation, AI integrates multi-omics data to systematically reveal the regenerative potential of MSC-EVs and provide new standards for batch release and QC in large-scale production [361]. For example, by analyzing key proteins or nucleic acids that AI predicts to be related to tissue regeneration, functional maps can be constructed to evaluate the therapeutic potential of MSC-EVs products as CQAs. Models such as support vector machines and graph neural networks can deeply analyze the complex signaling networks mediated by MSC-EVs, providing new insights into core mechanisms, such as immune modulation and angiogenesis [361]. Furthermore, AI-based analytical systems can even predict individual responses to MSC-EVs treatment by analyzing EV characteristics in patient body fluids, which lays the foundation for personalized regenerative therapies [353]. In conclusion, AI technology is playing a significant role in the standardization of MSC-EVs preparation, targeted functional optimization, and mechanism analysis. The deep integration of AI with large-scale MSC-EV production systems will effectively promote the translation of this advanced regenerative medicine strategy from the laboratory to clinical applications, achieving stable, efficient, and controllable industrialization goals.

#### Strategic framework for MSC-EV clinical translation across quality control, non-clinical studies and regulatory pathways

The clinical translation of MSC-EVs requires a well-defined strategic framework that integrates three fundamental pillars: comprehensive QC, systematic non-clinical studies, and clearly defined regulatory pathways. First, robust quality management systems must be implemented throughout the manufacturing process. This requires a thorough characterization of critical quality attributes to ensure product safety, consistency, and efficacy [362]. These attributes encompass identity verification, particle size distribution, concentration analysis, component profiling, purity assessment, and impurity detection. Identity is confirmed through the detection of positive surface markers, including CD9, CD63, and CD81, *via* flow cytometry or western blot. Crucially, the absence of negative markers such as Calnexin and GM130 must also be verified to rule out cellular or endoplasmic reticulum contamination [362]. Particle size distribution and concentration are quantitatively analyzed using nanoparticle tracking analysis or nanoflow cytometry, which reflects the correlation between particle size distribution and particle number [362]. Component profiling involves the qualitative and quantitative analysis of EV lipids, proteins, and nucleic acids *via* omics technologies. Purity is assessed by defining the particle-to-protein ratio and quantifying lipoprotein impurities, including Apolipoprotein A1 and Apolipoprotein B which are common process-related contaminants. Impurity detection identifies non-membranous particles and free molecules, such as nucleic acids, proteins, and lipids, that lack membrane integrity [362]. For engineered EVs, additional evaluations, including the identification of loaded therapeutic molecules and drug-loading parameters, are essential to guarantee product quality.

While these physicochemical parameters are essential for product consistency, they do not directly predict therapeutic efficacy. For therapeutic applications, establishing potency is paramount. Potency must be assessed based on the mechanism of action relevant to the intended clinical indication. This is typically achieved through multi-level evaluations that include molecular, cellular, and *in vivo* models to confirm dose-dependent efficacy. A robust potency assay is critical for batch-to-batch consistency and regulatory approval, as it directly links product quality to clinical outcomes [362]. Determining the ideal therapeutic dose remains a translational hurdle. A scoping review revealed substantial heterogeneity in dosage units across registered MSC-EV trials, including volume, total particle number, and protein mass, which complicates cross-study comparisons [363]. Optimal dosing should integrate nonclinical pharmacokinetic and pharmacodynamic data with early-phase dose-escalation trials, and it must be justified by disease-specific pathophysiology and the chosen administration route.

Second, comprehensive non-clinical validation should demonstrate therapeutic potential through rigorous pharmacokinetic and safety assessments in biologically relevant models [364–368]. These studies must characterize key parameters including biodistribution patterns, clearance rates, metabolic pathways, and biological half-lives. Additionally, a thorough evaluation of potential impacts on major physiological systems including respiratory, neurological, and cardiovascular functions is crucial. The assessment should address both the targeted toxicity resulting from active pharmaceutical ingredients carried by EVs and the broader safety considerations related to product characteristics and administration.

A critical concern for clinical translation is long-term safety and tumor-related risks. MSC-EVs are not living organisms and cannot replicate, which inherently eliminates the risk of abnormal cell differentiation or tumor formation associated with live cell therapies. However, because EVs are rich in bioactive molecules including non-coding RNAs and proteins with transformation potential, theoretical risks cannot be entirely dismissed. The Japanese Pharmaceuticals and Medical Devices Agency (PMDA) review emphasizes that when EVs are derived from immortalized cell lines, they may contain retrotransposon sequences that could theoretically transfer malignant phenotypes to normal cells, warranting careful evaluation of genomic stability during cell bank establishment. Therefore, long-term toxicity studies should monitor for potential tumor-promoting effects, particularly when using immortalized or long-term cultured cells for large-scale production [369].

In parallel with these long-term considerations, off-target effects represent another important safety dimension. For native MSC-EVs, off-target effects are considered minimal due to the inherent target specificity of their constituent molecules [369]. However, for engineered MSC-EVs such as those modified with targeting ligands or loaded with exogenous therapeutic molecules, engineering modifications may alter surface topology, biodistribution patterns, and cellular uptake. This could potentially lead to unintended organ accumulation or biological activity in non-target cells [362]. These risks warrant case-by-case evaluation, with particular attention paid to the safety of any introduced chemical or genetic modifications.

A further consideration relates to immunogenicity and immune-related risks. MSC-EVs are generally considered to have low immunogenicity due to their non-replicative nature and the absence of cellular components [362]. However, the PMDA review notes that when allogeneic human-derived EVs are administered repeatedly, immune responses may develop, potentially attenuating therapeutic efficacy or causing adverse reactions [369]. Additionally, impurities such as residual culture medium components or process-related contaminants may elicit allergic or inflammatory responses, which underscores the importance of rigorous purification and QC [369]. For engineered EVs carrying exogenous molecules, immunogenicity assessment becomes even more critical, as chemical modifications or foreign proteins may trigger unwanted immune activation [362].

Third, navigating the evolving regulatory landscape requires an understanding of the classification pathways established by major regulatory authorities [370–372]. China's Center for Drug Evaluation has classified EVs within the Advanced Therapy Medicinal Products framework, while the United States Food and Drug Administration regulates them as biological products requiring Investigational New Drug applications. Similarly, the European Union follows biological medicinal product regulations for EV-based therapies.

The synchronization of these interconnected elements including QC, non-clinical evidence, and regulatory strategy forms a solid foundation for translating MSC-EVs research into clinically viable therapies. This integrated approach ensures that product development progresses with clearly defined quality standards, comprehensively demonstrated biological activity, and full compliance with international regulatory requirements. Consequently, it effectively bridges the gap between laboratory research and clinical applications while maintaining the highest standards of patient safety and therapeutic potential.

#### Clinical trials

Currently, the number of clinical trials evaluating MSC-EVs is rapidly increasing, and they have become a primary focus of EV therapy research (Table 7). MSC-EVs are primarily isolated from various tissues, including the bone marrow, adipose tissue, neonatal tissues, and synovial fluid. More than half of the registered trials are designed as controlled studies, with most adopting randomization, double-blind, or triple-blind protocols. Early-phase trials primarily focus on safety assessments while also exploring preliminary therapeutic efficacy [363].

**Table 7:** Summary of representative clinical trials on MSC-EVs for regenerative applications

NCT number	Condition/disease	EV source	Sponsor	Phase	Brief summary
NCT05078385	Second-degree burns	Human BMMSCs	Aegle Therapeutics	Phase 1	Evaluates BMMSC-EVs for treating deep second-degree burns, aiming to enhance wound healing and reduce scarring.
NCT05243368	Diabetic skin ulcers	Unclear	Maimónides Biomedical Research Institute of Córdoba	Not Applicable	Assesses MSC-EVs for healing chronic skin ulcers, particularly in diabetics, by improving the regenerative microenvironment.
NCT05813379	Skin rejuvenation	Unclear	Isfahan University of Medical Sciences	Phase 1 Phase 2	Aims to slow skin aging using MSC-EVs, primarily by stimulating collagen production for cutaneous regeneration.
NCT05971342	Alveolar Bone Healing	Human UCMSCs	Fifth Affiliated Hospital of Guangzhou Medical University	Not Applicable	Tests gelatin sponge-loaded UCMSC-ApoVs for regenerating alveolar bone after mandibular third molar extraction.
NCT04270006	Periodontitis	Human ADMSCs	Beni-Suef University	Early Phase 1	Evaluates autologous ADMSC-EVs injected into periodontal pockets to assess their regenerative effect.
NCT05060107	Knee osteoarthritis	Unclear (allogeneic)	Francisco Espinoza	Phase 1	A Phase I trial assessing the safety of intra-articular injections of allogeneic MSC-EVs for knee osteoarthritis.
NCT05261360	Degenerative meniscal injuries	Human SFMSCs	Eskisehir Osmangazi University	Phase 2	Compares the efficacy of intra-articular SFMSC-EVs versus the parent MSCs in the same patient.
NCT05871463	Liver cirrhosis	Human UCMSCs	Research Institute for Gastroenterology and Liver Diseases	Phase 2	Evaluates the safety and efficacy of UCMSC-EVs as a potential therapy for decompensated liver cirrhosis.
NCT05787288	COVID-19	Unclear	First Affiliated Hospital of Wenzhou Medical University	Early Phase 1	Investigates the safety and efficacy of nebulized MSC-EVs for alleviating COVID-19-induced lung injuries alongside standard therapy.
NCT04213248	GVHD-related dry eye	Human UCMSCs	Zhongshan Ophthalmic Center, Sun Yat-sen University	Phase 1 Phase 2	Determines if UCMSC-EVs can alleviate dry eye symptoms in patients with chronic GVHD.

*MSC* mesenchymal stem cell, *EV* extracellular vesicle, *NCT number* national clinical trial number, *BMMSCs* bone marrow mesenchymal stem cells, *UCMSCs* umbilical cord mesenchymal stem cells, *ADMSCs* adipose-derived mesenchymal stem cells, *SFMSCs* synovial fluid-derived mesenchymal stem cells, *GVHD* graft-versus-host disease

In the dermatological field, several early-stage clinical studies have been initiated, including trials for second-degree burns (NCT05078385), diabetic skin ulcers (NCT05243368), and skin rejuvenation (NCT05813379) [373]. In the oral and maxillofacial region, one study evaluated the role of lyophilized MSC-ApoVs following the extraction of impacted mandibular third molars (NCT05971342) [374]. The results showed that these ApoVs remained stable when stored at 4°C for up to three months and significantly reduced hemostasis time and promoted alveolar bone regeneration by upregulating tripartite motif containing 71 (TRIM71) and activating the ERK signaling pathway. This was the first study to confirm the safety and efficacy of ApoVs as a cell-free therapy in oral tissue repair. In musculoskeletal diseases, trials have explored the potential of intra-articular MSC-EVs injections for treating OA (NCT05060107) and degenerative meniscal injuries (NCT05261360) [335]. In the respiratory system, COVID-19 and related acute respiratory distress syndrome have become research hotspots. One clinical study reported that a single intravenous infusion of BMMSC-EVs in critically ill COVID-19 patients significantly improved oxygenation. Specifically, the average arterial oxygen partial pressure to inspired oxygen fraction ratio (PaO_2_/FiO_2_) increased by 192%. The treatment also reduced inflammation markers and restored lymphocyte subpopulations. The infusion was associated with no treatment-related adverse events, suggesting potential for alleviating respiratory failure and immune dysregulation [375]. In immunological and hematological disorders, graft-versus-host disease (GVHD) has been a key research focus. A case report showed that after four treatments with placenta MSC-EVs, a 39-year-old chronic GVHD patient experienced improved skin pigmentation, ulcer healing, and reduced inflammation markers, without side effects [376]. Further studies have formulated the MSC-EVs into eye drops, which were applied to 14 patients with refractory GVHD-related dry eye (NCT04213248). The results showed reduced corneal damage, improved tear film stability, and increased tear secretion, leading to significant improvements in patient symptoms and quality of life [377].

Although current clinical results demonstrate the potential of MSC-EVs in treating various diseases, challenges remain, including a lack of standardization in EV preparation and characterization, small sample sizes, and significant heterogeneity across studies [378–380]. Notably, a comprehensive analysis of registered trials found that none of the 49 MSC-EV studies reported sufficient details to satisfy the MISEV2018 guidelines, which underscores the need for standardized reporting [363]. Future trials should mandate comprehensive characterization data, including isolation protocols, particle size, concentration, surface marker profiles, purity, and mechanism-based potency assays. This will enable meaningful data aggregation and accelerate clinical translation. Future research should also focus on large-scale, standardized EV production and the conduct of multi-center, large-sample randomized controlled trials to further explore the underlying mechanisms of action.

## Conclusions

MSC-EVs have established themselves as a transformative acellular platform in regenerative medicine through their immense therapeutic potential for orofacial, barrier, musculoskeletal, and visceral tissue repair. This versatility arises from their inherently low immunogenicity, multifaceted biological activity, and expansive potential for functional engineering. The primary advantage of these vesicles is their ability to deliver complex bioactive cargoes consisting of proteins, lipids, and nucleic acids that collectively orchestrate immune responses, angiogenesis, anti-fibrotic remodeling, and cell death, while promoting barrier restoration and endogenous stem cell activation. Despite significant preclinical successes and encouraging results from early clinical investigations, widespread translation remains constrained by inherent vesicle heterogeneity, insufficient targeting specificity, and bottlenecks in large-scale manufacturing and QC standardization. Realizing the full clinical promise of MSC-EVs requires a strategic integration of injury microenvironment analysis, functional engineering, and intelligent manufacturing frameworks to better align mechanistic insights with product development. As high-throughput sequencing, AI, and advanced biomanufacturing systems continue to evolve, MSC-EV-based therapies are poised to lead regenerative medicine into a new era of precision and individualized therapy.
